# Targeting METTL3 mitigates venetoclax resistance via proteasome-mediated modulation of MCL1 in acute myeloid leukemia

**DOI:** 10.1038/s41419-025-07560-w

**Published:** 2025-04-01

**Authors:** Chang-qing Jiao, Chen Hu, Meng-hua Sun, Yan Li, Chao Wu, Fei Xu, Lei Zhang, Fu-rong Huang, Jun-jie Zhou, Ji-fei Dai, Min Ruan, Wen-chao Wang, Qing-song Liu, Jian Ge

**Affiliations:** 1 Department of Hematology, the First Affiliated Hospital of Anhui Medical University, Hefei 230022, China & Anhui Medical University, Hefei, 230032 China; 2 Anhui Province Key Laboratory of Medical Physics and Technology, Institute of Health and Medical Technology, Hefei Institutes of Physical Science, Chinese Academy of Sciences, Hefei, 230031 China; 3 Hefei Cancer Hospital, Chinese Academy of Sciences, Hefei, 230031 China

**Keywords:** Acute myeloid leukaemia, Drug development

## Abstract

Venetoclax, a selective BCL2 inhibitor, is extensively utilized in clinical settings for the treatment of acute myeloid leukemia (AML). However, its efficacy is often compromised by the development of drug resistance. Hence, identification of potential venetoclax combination treatment strategies is imperative to overcome this acquired resistance. In this study, we discovered that inhibition of METTL3 can synergistically enhance the anti-leukemic efficacy of venetoclax, and is capable of overcoming venetoclax resistance in in vivo experiments and various venetoclax resistance models. Mechanistic study revealed that STM2457 augmented venetoclax activity by downregulating MCL1 and MYC, thereby increasing apoptosis in leukemia cells induced by venetoclax. Further investigation demonstrated that STM2457 promotes the ubiquitination and subsequent protein degradation of MCL1 primarily through pharmaceutically targeting METTL3. Moreover, through molecular docking-based virtual screening, we identified isoliquiritigenin as a potential novel small molecule natural product targeting METTL3, which exhibited potential effects as an anti-leukemic agent.

## Introduction

Acute myeloid leukemia (AML) is a diverse and aggressive disease characterized by the malignant growth of hematopoietic cells, primarily myeloblasts, which impedes normal blood cell formation [1]. Despite advances in high-dose chemotherapy and stem cell transplants, many AML cases remain incurable with a 5-year survival rate of less than 20% in the elderly [2–4]. AML primarily affects older adults with a median diagnosis age of 68 [5]. Many elderly patients cannot tolerate standard intensive chemotherapy, pressing for more effective and well-tolerated treatment regimens.

Recent advancements in leukemia treatment include targeting the mitochondrial apoptotic pathway in cancer cells. Overexpression of BCL2 in AML leads to impaired apoptosis and drug resistance [6, 7]. Thus, small molecule inhibitors of BCL2 can effectively induce AML apoptosis [8]. Venetoclax, a selective BCL2 inhibitor, has been recommended in co-therapies with hypomethylating agents for patients unsuitable for intensive chemotherapy [9]. However, venetoclax monotherapy shows limited efficacy with a remission rate of just 19% [10]. Around 73% of AML patients responded to the venetoclax and azacitidine combination, but over half of the patients eventually relapsed [11]. In relapsed/refractory patients receiving similar treatment combinations, the remission rate was just 21% with a median survival of 3 months [12]. These data indicate both intrinsic and acquired resistance to venetoclax, and thus combining venetoclax with other drugs may help overcome this resistance or increase its efficacy. We previously discovered that dexamethasone enhances venetoclax efficacy by reducing mitochondrial transfer between bone marrow mesenchymal stem cells and AML cells [13]. Other teams found Myeloid Cell Leukemia 1(MCL1) protein stability was identified as a key factor of venetoclax resistance in AML [14, 15]. In-depth understanding of the interactions between proteins such as MCL1 and venetoclax may help pinpoint adjunct medications for co-therapy.

METTL3 serves as a central methyltransferase within the N6-methyladenosine (m6A) modification system [16], functioning as the m6A “writer” implicated in both the initiation and maintenance of AML [17, 18]. STM2457, a small-molecule inhibitor against METTL3 developed by Storm Therapeutics, has exhibited antiproliferative effects in AML [19]. Additionally, several studies have indicated that METTL3 is associated with chemoresistance in AML, and STM2457 can overcome chemoresistance through degradation of ITGA4 mRNA [20]. Nevertheless, while STM2457 has promising potential for clinical therapy, the possible interactions between METTL3 inhibitors and venetoclax have not been previously explored.

In this study, we demonstrated that inhibition of METTL3 by STM2457 downregulates MCL1 and MYC, which were key factors associated with venetoclax resistance. Combination of STM2457 and venetoclax showed synergistic anti-leukemic efficacy by increasing cell apoptosis in both AML cell lines and primary patient samples. Further, this combined effect was recapitulated in the AML cell xenograft model and multiple venetoclax resistance models. In addition, we found that STM2457 treatment upregulates E3 ligase FBXW7 and leads to the degradation of MCL1 protein via the FBXW7-dependent ubiquitin-proteasome system. The mRNA of FBXW7 was m6A modified by METTL3 and then recognized by m6A reader YTHDF2, which weakens FBXW7 mRNA stability, resulting in FBXW7 protein reduction. Finally, we found a novel METTL3 inhibitor, a natural product called isoliquiritigenin, with potential anti-leukemia activity.

## Materials and methods

### Patients and healthy donors

Bone marrow or peripheral blood samples from AML patients or healthy donors were used with informed consent and approval from the Ethical Committee of the First Affiliated Hospital of Anhui Medical University. Mononuclear cells were isolated via density gradient separation per manufacturer’s instructions (Ficoll, Tbdscience). Patient characteristics are detailed in Supplementary Tables S1. Primary AML cells were cultured in specific medium for human acute myeloid leukemia cells (PRS-AMLM, PRECEDO). Healthy donor PBMCs were cultured in RPMI1640 (KeyGEN) with 10% fetal bovine serum (FBS, VivaCell), 1% NEAA (Gibco, 11140050), and 1% sodium pyruvate (Gibco, 11360070).

### RNA stability

Actinomycin D (5 μg/mL) was used to halt mRNA transcription in cultured cells for RNA stability assays. Cells were collected at various time points post-treatment, and MCL1 mRNA levels were quantified using qRT-PCR.

### RT-qPCR and RIP-qPCR

1) RT-qPCR was carried out as previously described [21]. Primers were listed in Supplementary Table S2. Results were analyzed using the 2^−(ΔΔCt)^ method.

2) 1.5 µg of METTL3 or YTHDF2 antibody, along with control IgG, was conjugated to protein G magnetic beads (MCE) by incubating for 4 h at 4 °C. Ten million cells were harvested, washed with cold PBS, and UV crosslinked at 254 nm. Cells were lysed in 1 mL RIP buffer with inhibitors for 10 min. 10% of the lysate was saved as input, and the remaining lysate was incubated with protein A/G magnetic beads at 4 °C overnight with rotation. Immunoprecipitated RNAs were recovered by TRIzol extraction and analyzed by RT-qPCR.

### Molecular docking

The details were provided in Supplementary Materials and Methods. The crystal structure of METTL3 was obtained from the Protein Data Bank (PDB ID: 5K7U). Water molecules and the original ligand were manually removed by using PyMol software(version 1.8). Prediction of the binding pose of isoliquiritigenin(provided by Topscience) was carried out by Autodock (version 4.2.6) [22].

### M6A dot blot assay

Total RNA was extracted and denatured at 95 °C for 3 min. 400 ng of RNA was then applied dropwise onto nylon (Solarbio) or NC (Millipore) membranes in duplicate. The membranes were UV cross-linked for 1 hour, blocked with 5% nonfat milk, and incubated overnight with an m6A antibody. Dot blot images were immobilized on NC membrane and immunolabelled with HRP-conjugated secondary antibody, with methylene blue staining used as a loading control.

### Statistical analysis

Statistical analysis was performed using GraphPad Prism8.0 and R software (version 4.2.2). Student’s *t* test or one-way ANOVA was used to assess the statistical significance. Survival curves were assessed by the Kaplan–Meier method and compared by the log-rank test. Statistical significance was defined as follows: *P < 0.05, **P < 0.01, and ***P < 0.001.

## Results

### STM2457 synergizes with venetoclax to inhibit AML cell growth

**Fig. 1:**
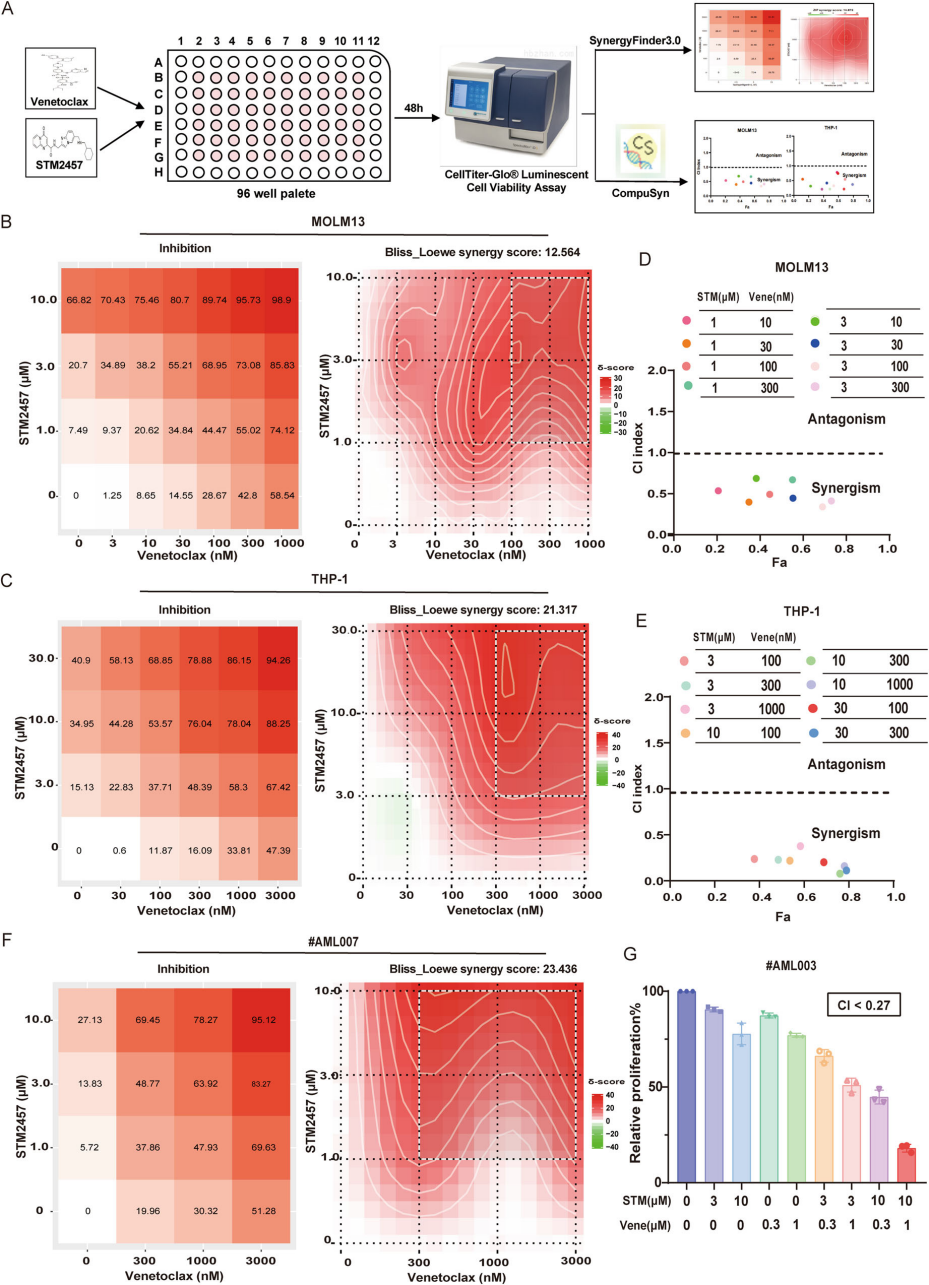
STM2457 synergizes with venetoclax in AML cells. **A** Schematic illustration for exploring drug synergistic effects of venetoclax and STM2457 combination in AML cells. **B**, **C** Cell viability of Molm13 and THP-1 cells treated with STM2457, venetoclax, or their combination at different concentrations for 48 h was assessed using the CellTiter-Glo® Luminescent Cell Viability Assay. Synergy was assessed by the SynergyFinder 3.0(https://synergyfinder.fimm.fi). **D**, **E** Combination Index (CI) of venetoclax and STM2457 in Molm13, THP-1, and primary AML cells was calculated using compusyn software. CI values indicate synergistic (CI < 1), additive (CI = 1), or antagonistic (CI > 1) effects. **F**, **G** Cell viability in primary AML cells treated with venetoclax, STM2457, or their combination for 48 h was assessed using the CellTiter-Glo assay.

To determine whether STM2457 exhibits a synergistic effect with venetoclax on the proliferation of AML cells, following the flowchart as shown in Fig. 1A, we exposed Molm13 and THP-1 cells to varying concentrations of STM2457, venetoclax, or their combination for 48 h. We assessed the synergistic effect of the two drugs by two computational methods using the SynerFind 3.0 web tool [23], and found that STM2457 and venetoclax have a significant synergistic effect in both Molm13 and THP-1 cells (Fig. 1B, C), with Combination Index(CI) ranging from 0.33 to 0.69 for Molm13 and from 0.08 to 0.38 for THP-1 (Fig. 1D, E). The doses demonstrating the most pronounced synergistic effect were subsequently selected for further study (Supplementary Tables S3 and S4). The anti-proliferation effect of STM2457 and venetoclax on AML cells was then evaluated by EdU assay, which showed the combination of STM2457 and venetoclax exhibited better anti-proliferation activity compared to either treatment alone (Supplementary Fig. S1A-B). Furthermore, we validated the synergistic effect of STM2457 with venetoclax in primary AML samples, where the combination showed stronger anti-leukemic effect compared to monotherapy (Fig. 1F, G). In addition, the cytotoxicity was evaluated on healthy donor PBMCs, healthy stromal HS-27A cells, and bone marrow mononuclear cells (BMMNCs), which showed that the combination of STM2457 and venetoclax only exhibited slight inhibitory effects at high dosages (Supplementary Fig. S1C–E). Together, these results suggested that STM2457 is a potential agent for combination strategy with venetoclax to enhance anti-AML efficacy.

### STM2457 enhances the apoptosis of AML cells induced by venetoclax through suppression of MCL1 and MYC expression

**Fig. 2:**
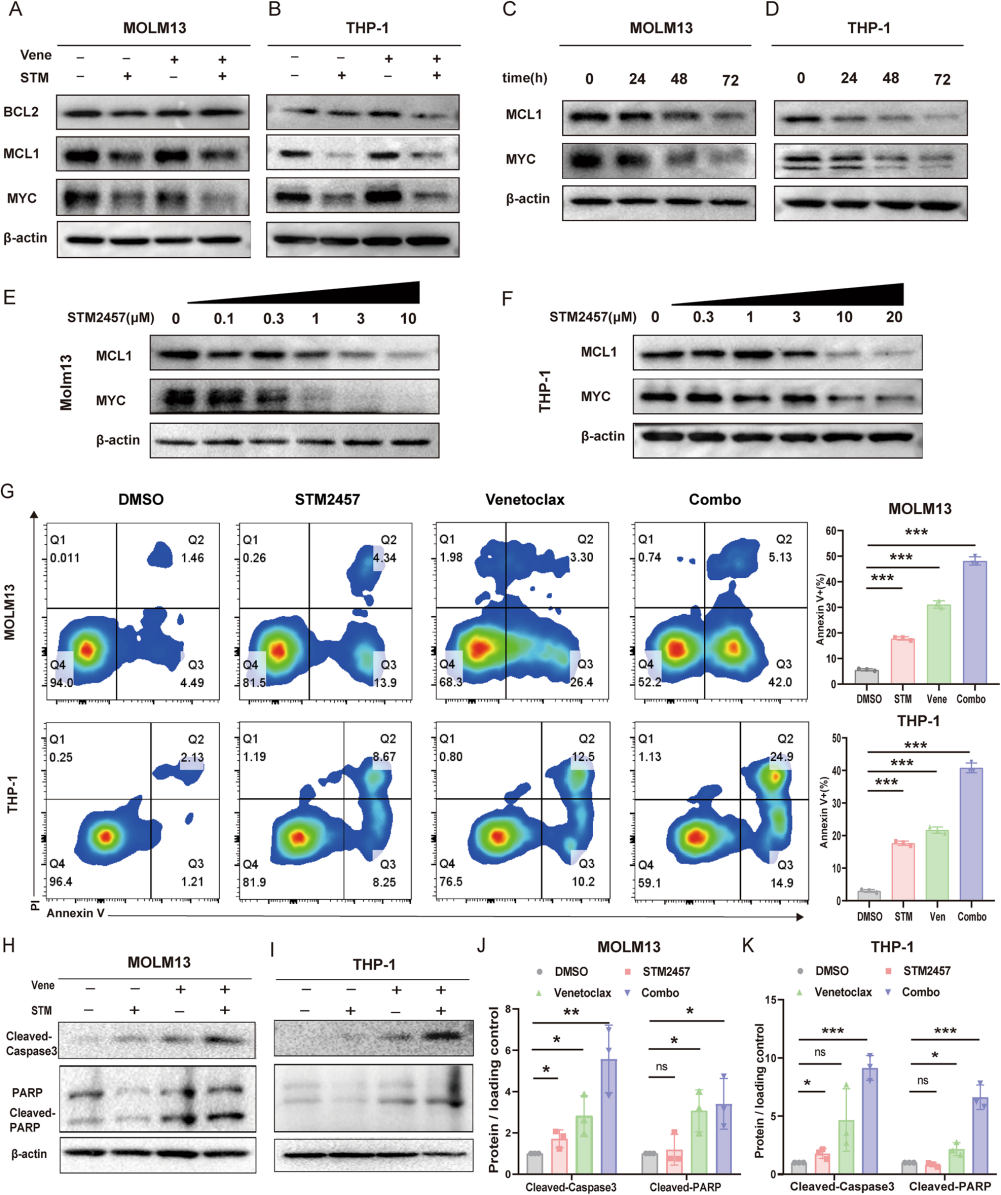
STM2457 enhances the AML cell apoptosis induced by venetoclax through suppression of MCL1 and MYC. **A**, **B** Protein expression of BCL2, MCL1, and MYC in Molm13 and THP-1 cells treated with venetoclax(100 nM, 300 nM), STM2457(3 μM, 10 μM), or combination as analyzed by Western blotting. **C**, **D** Western blot analysis of Molm13 and THP-1 cells treated with STM2457(3 μM, 10 μM) at different times. **E**, **F** Western blot analysis of Molm13 and THP-1 cells exposed to STM2457 at the indicated concentration for 48 h. **G** Apoptosis assay of Molm13 and THP-1 cells treated with venetoclax (100 nM, 300 nM), STM2457 (3 μM, 10 μM), or their combination for 48 hours. Apoptosis was measured by flow cytometry, and percentages were averaged from three independent experiments. **H**–**K** Western blot analysis of cleaved-caspase3 and cleaved-PARP in Molm13 and THP-1 cells with venetoclax and STM2457 alone or indicated combinations for 48 h. β-actin was used as loading control. Data were expressed ±SEM. All by Student’s *t* test. *p < 0.05; **p < 0.01;***p < 0.001;ns not significant.

We then aimed to investigate the mechanism of the synergistic effects of STM2457 with venetoclax. As previously reported, the upregulation of MCL1 and MYC after venetoclax exposure is partially responsible for the insensitivity to venetoclax [24, 25]. Therefore, we first evaluated the protein levels of MCL1 and MYC in THP-1 and Molm13 cells after drug treatments. Our results showed that the combo treatment significantly reduced the levels of both MCL1 and MYC in both AML cell lines (Fig. 2A, B). Furthermore, we validated the reduction of MCL1 and MYC proteins induced by STM2457 was both time- and concentration-dependent on Molm13 and THP-1 cells (Fig. 2C–F). Meanwhile, knockdown of METTL3 also led to a decrease in the protein levels of MCL1 and MYC (Supplementary Fig. S2A), and overexpression of METTL3 augmented the two proteins (Supplementary Fig. S2B).

Next, we evaluated whether STM2457 could enhance the pro-apoptotic effects of venetoclax through the dual suppression of MYC and MCL1. After 48 hours of treatment with STM2457 and venetoclax in Molm13 and THP-1 cells, significant alterations in cell morphology were observed in the combination treatment group compared to the monotherapy groups (Supplementary Fig. S3A). Notably, the combination treatment exhibited a pronounced additive effect in inducing apoptosis and reducing the mitochondrial membrane potential, as evidenced by Annexin V/PI staining (Fig. 2G) and JC-1 staining (Supplementary Fig. S3B-C). To further confirm that STM2457 enhances venetoclax-induced apoptosis in AML, we analyzed apoptosis-related proteins, including PARP and cleaved Caspase-3 by Western blotting, which demonstrated increased cleavage of PARP and Caspase-3 in the combination treatment group (Fig. 2H–K). Collectively, these results indicate that STM2457 synergizes with venetoclax to enhance AML cell apoptosis by simultaneously decreasing the expression of MCL1 and MYC.

In addition, the protein levels of MCL1 in AML cell lines showed a statistically significant negative correlation with the GI_50_ values of STM2457 (r = -0.684, *P* = 0.02), suggesting that MCL1 levels may serve as a potential biomarker for STM2457 efficacy in AML (Supplementary Fig. S4A–C). Together, our results indicate that STM2457 enhances anti-tumor effects of venetoclax by decreasing the expression of both MCL1 and MYC.

### The combination of STM2457 and venetoclax inhibits xenograft growth in vivo

**Fig. 3:**
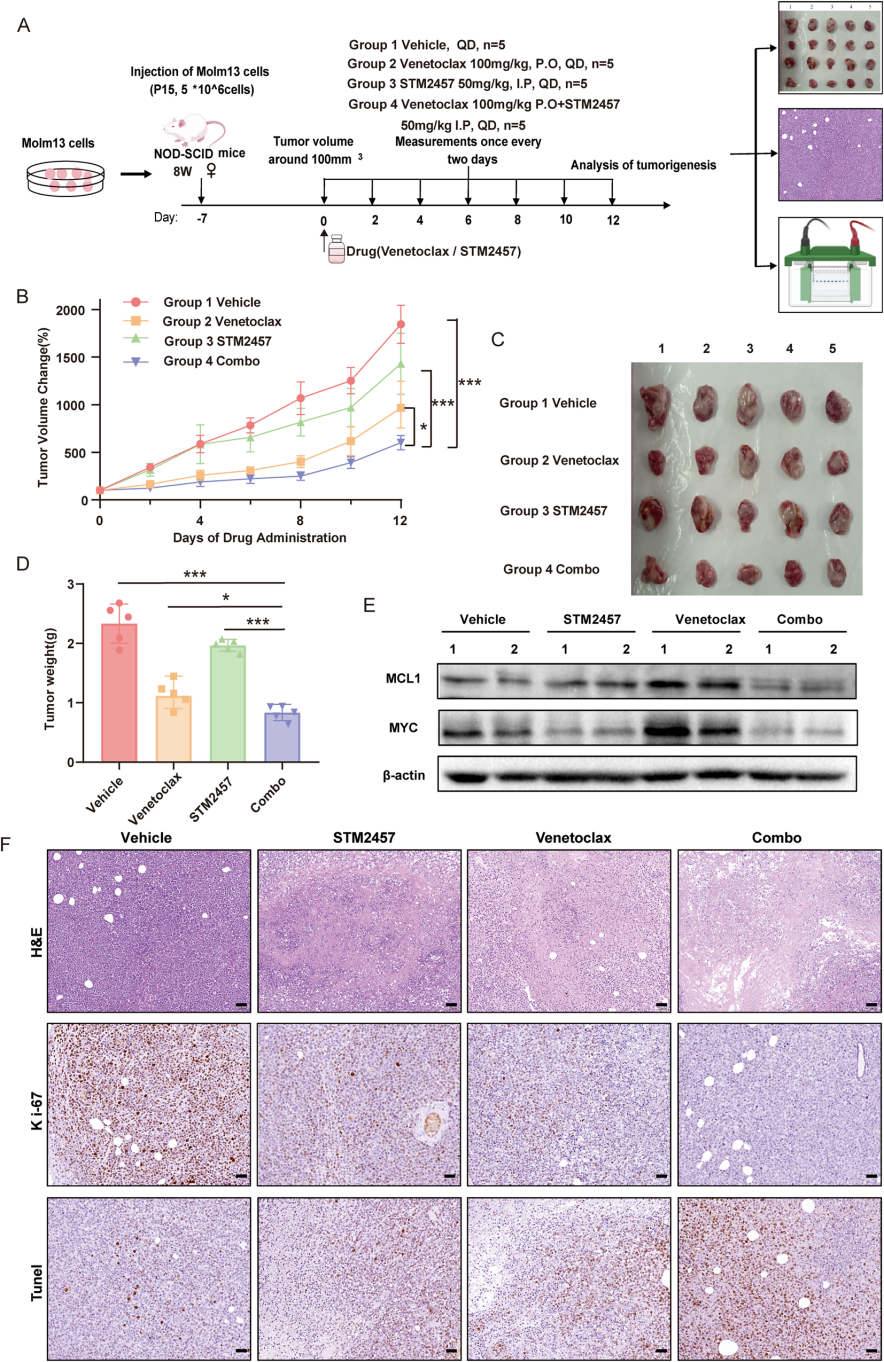
STM2457 enhances the anti-leukemic efficacy of venetoclax in an AML xenograft (Molm13) model. **A** Schematic representation of the AML cells in vivo study design. **B** Tumor growth curves recorded in the xenograft mice treated with indicated reagents. **C** Images of subcutaneous xenograft tumors at the endpoints treated with indicated reagents. **D** Measurement of tumor weight in each group (*n* = 5) after being treated with indicated reagents. **E** The MCL1 and MYC protein levels in tumor tissues were detected by Western blotting. **F** Representative images of IHC staining of Ki-67 and TUNEL in the xenograft mice. Scale bars: 50 μm. Results in the graphs are expressed as means ± SD. *p < 0.05, **p < 0.01, ***p < 0.001, ns not significant.

To examine the effect of the combination of venetoclax and STM2457 in a more clinically relevant model, we constructed a xenograft mouse model (Fig. 3A). The combination significantly decreased tumor volume and tumor weight (Fig. 3B–D). Western blot analysis confirmed that STM2457 decreased MCL1 and MYC expression, consistent with our in vitro findings (Fig. 3E). In addition, we observed a marked decrease in Ki-67 protein level and an increase in TUNEL staining in the tumors from the combination group (Fig. 3F). In addition, as shown in the flow chart (Supplementary Fig. S5A), we also engrafted Molm13 cells into NCG mice to establish a Molm13 cell derived mouse model. This model was treated with vehicle, STM2457, venetoclax and combination respectively. When the mice in the vehicle group were on the verge of death, they were euthanized. Flow cytometry analysis of human CD45 positive leukemia cells showed that compared with single agent groups, the combination group significantly reduced the tumor burden in both the bone marrow and PBMCs (Supplementary Fig. S5B-C). Overall, the administration of STM2457 in combination with venetoclax demonstrated enhancement of anti-tumor efficacy.

### STM2457 promotes the ubiquitin-mediated proteasomal degradation of MCL1 by FBXW7 in AML cells

MYC has been reported as a target gene of METTL3, and inhibition of METTL3 by STM2457 decreased the m6A modification on MYC mRNA, resulting in a decrease in MYC translation [19]. However, the detailed mechanism underlying the decrease of MCL1 by STM2457 has not been explored. To elucidate the mechanism by which STM2457 affects MCL1, we first employed RT-qPCR to assess MCL1 mRNA levels and observed no significant changes after STM2457 treatment, METTL3 knockdown, and METTL3 overexpression in Molm13 and THP-1 cells (Supplementary Fig. S6). Subsequently, by RIP-qPCR and mRNA stability assays, we found that MCL1 mRNA did not interact with METTL3 protein, and there was no significant change in MCL1 mRNA stability after STM2457 treatment (Supplementary Fig. S7). These results indicated that METTL3 may regulate MCL1 through an indirect mechanism.

**Fig. 4:**
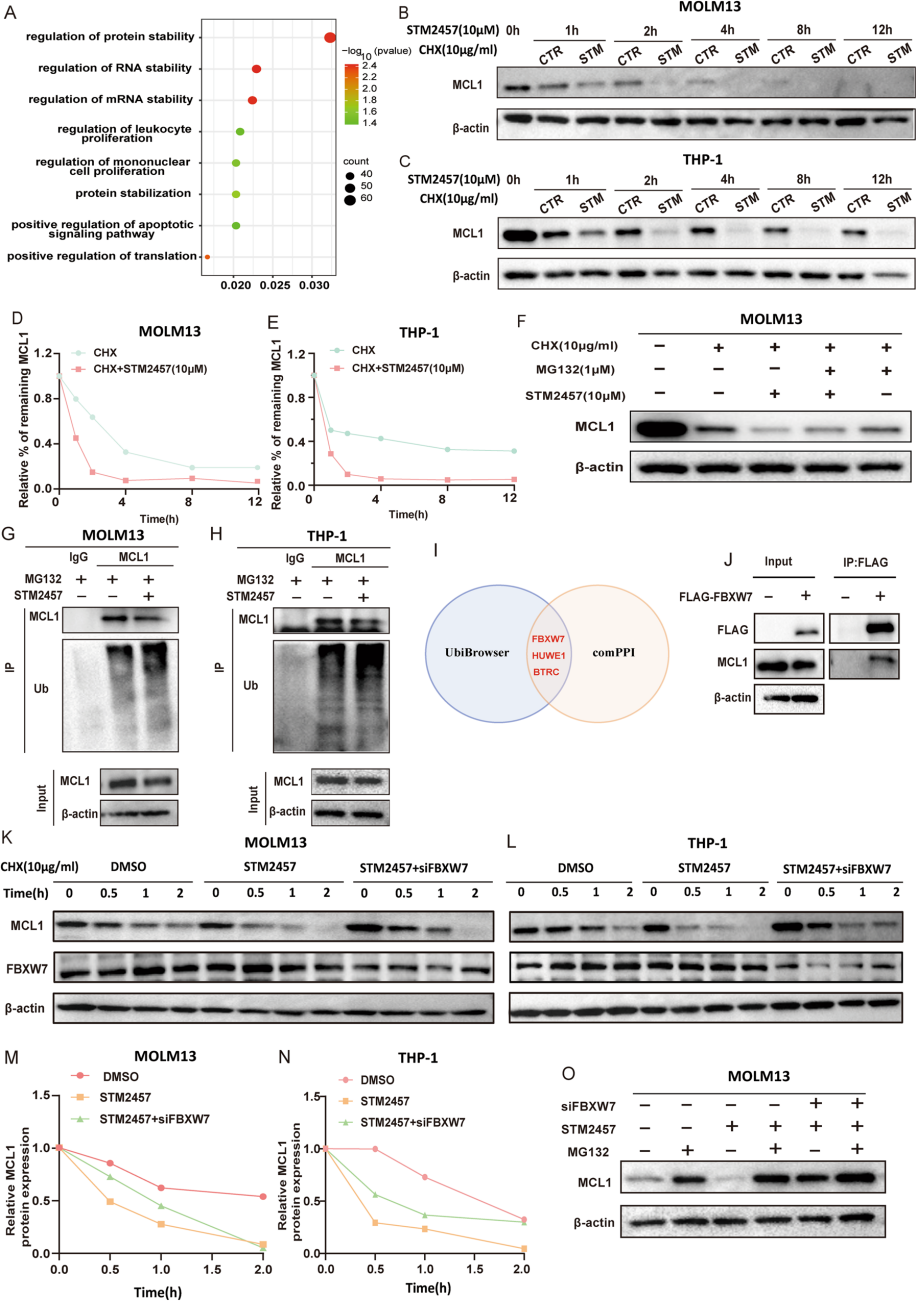
STM2457 increases MCL1 ubiquitination and degrades MCL1 protein via E3 ligase FBXW7. **A** GSEA enrichment analysis of Molm13 cells treated with or without STM2457 (ENA: PRJEB41662). **B**–**E** Effects of STM2457 on MCL1 protein degradation in Molm13 (**B**) and THP-1 (**C**) cells. Cells were treated with cyclohexane (CHX, 10 μg/ml) at indicated times. Relative MCL1 protein levels were recorded in (**D**) and (**E**). **F** Effects of MG132 treatment on MCL1 expression in Molm13 cells. Molm13 cells were pretreated with or without CHX (10 μg/ml), MG132(1 μM), and STM2457(10 μM) for 1 h. **G**, **H** The interaction between ubiquitin and MCL1 was assessed using Co-IP assay, and MCL1 was used as IP protein. **I** Overlapping analysis of proteins identified by UbiBrowser and ComPPI. The E3 ubiquitin ligase of MCL1 was predicted using the UbiBrowser website (http://ubibrowser.bio-it.cn/ubibrowser_v3/). The proteins that may potentially interact with MCL1 have been identified by ComPPI(https://comppi.linkgroup.hu/). **J** FLAG-FBXW7 plasmid was transfected in 293T for 48 h. Then Co-IP assay was used to assess the interaction between MCL1 and FBXW7. **K**–**N** Molm13 and THP-1 cells were transfected with FBXW7 siRNA, with or without STM2457 treatment, followed by CHX (10 μg/ml) exposure for various durations. MCL1 and FBXW7 protein levels were analyzed via Western blotting, and relative MCL1 levels were documented in (**M**) and (**N**). **O** After FBXW7 silencing and STM2457 treatment, Molm13 cells were co-cultured with or without MG132 (1 μM) for 4 h, and MCL1 protein expression was assessed by Western blotting.

We then performed enrichment analysis on the published dataset (ENA: PRJEB41662) and found that Molm13 cells treated with STM2457 were mainly enriched for genes involved in both RNA stability and protein stability (Fig. 4A). Therefore, we analyzed the stability of MCL1 protein using CHX chase assay. The results showed that STM2457 accelerated protein degradation of MCL1 (Fig. 4B–E). Additionally, MG132 pretreatment delayed the degradation of MCL1 protein (Fig. 4F and Supplementary Fig. S8A), suggesting that STM2457 may induce MCL1 protein degradation through the ubiquitin-proteasome pathway. To confirm this, we performed a ubiquitination assay via immunoprecipitation, which showed that the ubiquitination of immunoprecipitated MCL1 was significantly elevated in Molm13 and THP-1 cells treated with STM2457 (Fig. 4G, H). Taken together, these data suggest that STM2457 disrupts MCL1 stability by inducing its proteasomal degradation.

We further analyzed MCL1 ubiquitination-related proteins using ComPPI (https://comppi.linkgroup.hu/) (Supplementary Fig. S8B) [26] and UbiBrowser website (http://ubibrowser.bio-it.cn/ubibrowser_v3/) (Supplementary Fig. S8C) [27]. Overlapping analysis predicted that MCL1 might be ubiquitinated by E3 ubiquitin ligases FBXW7, HUWE1, and BTRC (Fig. 4I). Real-time qRT-PCR analysis revealed that FBXW7 mRNA levels increased after exposure to STM2457 in a dose-dependent manner, with no significant change in the mRNA levels of HUWE1 and BTRC (Supplementary Fig. S8D). In addition, the protein level of FBXW7 also increased upon STM2457 treatment, which showed a negative correlation to MCL1 levels (Supplementary Fig. S8E-F). Meanwhile, transfection with FBXW7 siRNA resulted in marked downregulation of FBXW7 and an upregulation of MCL1 in Molm13 and THP-1 cells (Supplementary Fig. S8G-H). Furthermore, Co-IP assays demonstrated a significant interaction between MCL1 and FBXW7 (Fig. 4J).

To investigate whether FBXW7 participates in the STM2457-induced MCL1 degradation in AML, we used RNA interference and found that knockdown of FBXW7 slowed the degradation of MCL1 by STM2457 in Molm13 and THP-1 cells (Fig. 4K–N). Moreover, treatment with MG132 further enhanced the increase of MCL1 mediated by FBXW7 siRNA in AML cells after STM2457 treatment (Fig. 4O and Supplementary Fig. S8I). Based on these results, we confirmed that in AML cells, STM2457 promotes MCL1 ubiquitination and proteasomal degradation through the upregulation of E3 ligase FBXW7. Notably, FBXW7 is a critical tumor suppressor of human cancers [28]. We also investigated the impact of FBXW7 expression on the prognosis of AML patients and found that the elevated levels of FBXW7 expression were associated with improved prognostic outcomes (Supplementary Fig. S8J).

### METTL3 regulates FBXW7 expression in a YTHDF2-dependent manner in AML cells

**Fig. 5:**
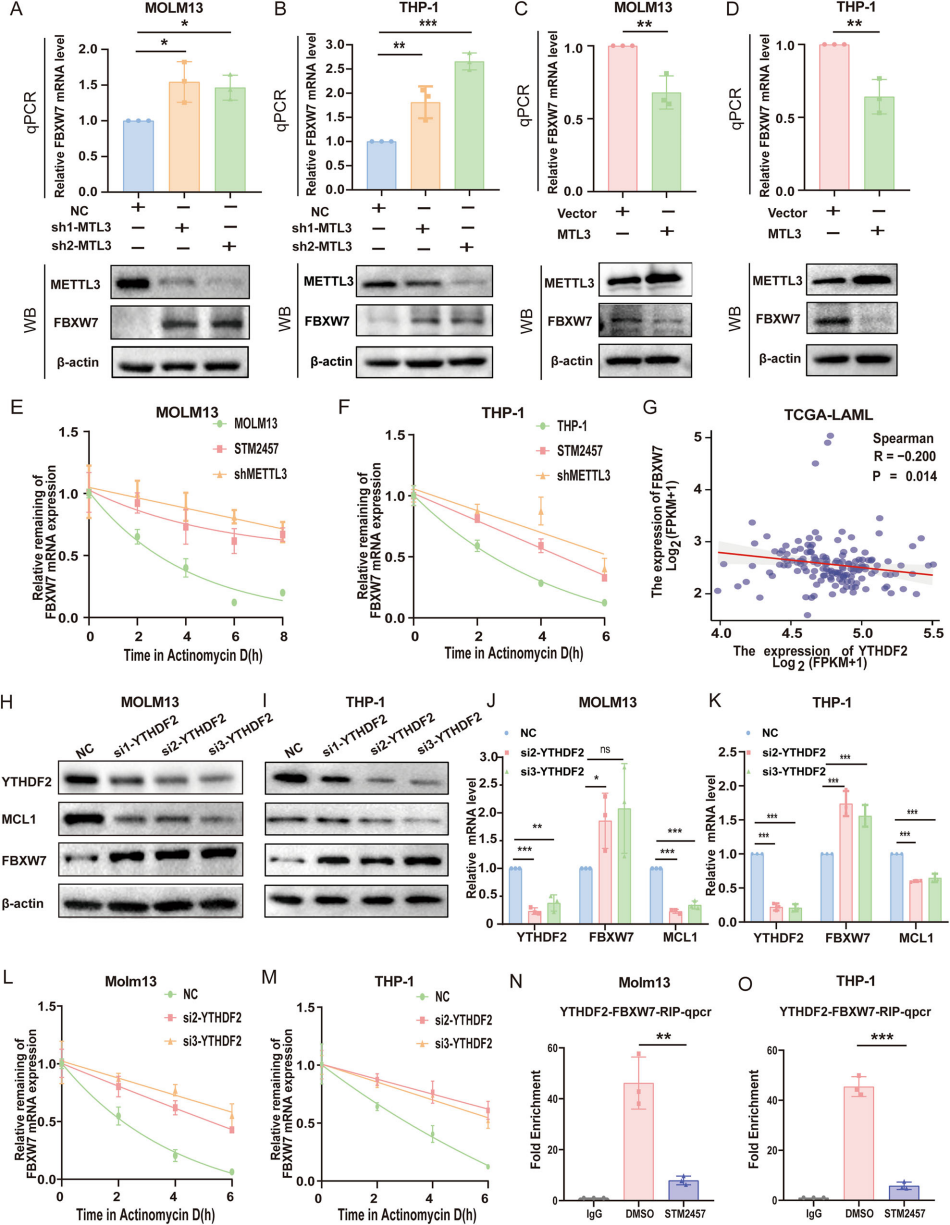
STM2457 reduced FBXW7 mRNA degradation through a METTL3 -YTHDF2-FBXW7-dependent pathway. **A**, **B** FBXW7 mRNA and protein levels in Molm13 or THP-1 cells transduced with lentivirus either expressing control(NC) or METTL3 shRNA(sh1 and sh2) were detected by RT- qPCR and western blotting, respectively. **C**, **D** FBXW7 mRNA and protein levels in Molm13 and THP-1 cells that were transfected with empty or METTL3-overexpressing vectors were detected by RT-qPCR and Western blotting, respectively. **E**, **F** RT-qPCR analysis of FBXW7 after actinomycin D treatment in shMETTL3, STM2457, and control Molm13 and THP-1 cells. Effects of METTL3 KD on FBXW7 mRNA degradation in Molm13 and THP-1 cells. Cells were treated with Actinomycin D at indicated times. G The correlation between FBXW7 and YTHDF2 in RNA levels in TCGA-LAML cohort. **H**, **I** Western blot analysis of YTHDF2, MCL1 and FBXW7 protein in Molm13 and THP-1 cells with YTHDF2 silencing. **J**, **K** The mRNA levels of YTHDF2, MCL1, and FBXW7 after YTHDF2 silencing in Molm13 and THP-1 cells were confirmed by RT-qPCR. **L**, **M** RT-qPCR analysis of FBXW7 after actinomycin D treatment in YTHDF2 siRNAs or Control Molm13 and THP-1 cells. **N**, **O** Anti-YTHDF2 RIP-qPCR test the interaction between FBXW7 mRNA and YTHDF2 in Molm13 and THP-1 cells. Data are shown as mean ± SD, *p < 0.05, **p < 0.01, ***p < 0.001, ns not significant, Student’s *t* test, one-way ANOVA analysis.

It has been reported that m6A modification of FBXW7 mRNA by METTL3 upregulates FBXW7 protein levels in lung adenocarcinoma [29]. Conversely, we found that the mRNA and protein levels of FBXW7 in AML cells were elevated following METTL3 knockdown and decreased by METTL3 overexpression (Fig. 5A–D). In addition, we found increased FBXW7 RNA stability with both METTL3 knockdown and STM2457 treatment in Molm13 and THP-1 (Fig. 5E, F). These results indicated that m6A-modified FBXW7 mRNA may be recognized by different m6A readers in AML, thereby affecting its stability. As YTHDF2 is a well-known m6A reader that affects mRNA degradation, we investigated whether YTHDF2 is the reader for FBXW7 mRNA in AML. First, we found that YTHDF2 mRNA level was negatively correlated with that of FBXW7 in AML samples from the TCGA database (Fig. 5G). As the results showed, both mRNA and protein levels of FBXW7 were elevated by knockdown of YTHDF2 in Molm13 and THP-1 cells (Fig. 5H–K), which was accompanied by increased FBXW7 RNA stability (Fig. 5L-M). Furthermore, RIP-qPCR confirmed the binding of FBXW7 mRNA to YTHDF2, and STM2457 treatment decreased the association of FBXW7 mRNA with YTHDF2 (Fig. 5N, O, Supplementary Fig. S9A-B), suggesting that YTHDF2 recognizes m6A-modified FBXW7 mRNA, affecting its mRNA stability.

Taken together, our data showed that in AML, FBXW7 mRNA is regulated by METTL3-YTHDF2 axis, which accelerates the degradation of FBXW7 mRNA. Inhibition of METTL3 by STM2457 enhances the stability of FBXW7 mRNA, subsequently increasing the ubiquitination and degradation of MCL1.

### STM2457 effectively mitigates resistance to venetoclax

**Fig. 6:**
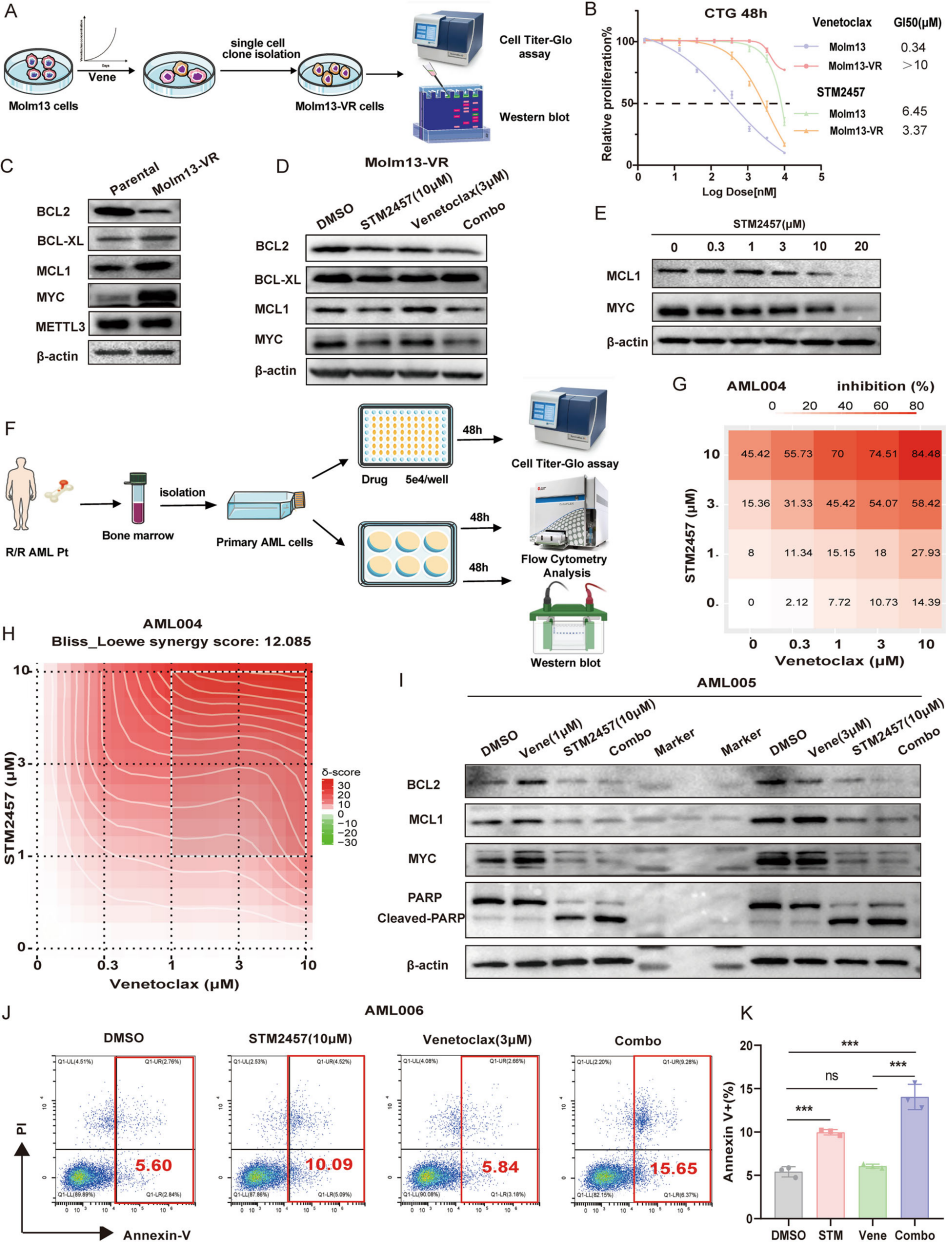
STM2457 effectively mitigates resistance to venetoclax in different AML models. **A** Diagram of generating venetoclax-resistant Molm13 cells: Molm13 cells were cultured with increasing doses of venetoclax. **B** Anti-proliferation effects of venetoclax and STM2457 on Molm13 and Molm13-VR cells were assessed using the CellTiter-Glo assay. **C** Western blot analysis of BCL2, BCL-XL, MCL1, MYC, and METTL3 protein levels of Molm13 parental and Molm13-VR cells. **D** Protein expression of BCL2, BCL-XL, MCL1, and MYC in Molm13-VR cells treated with venetoclax(3 μM), STM2457(10 μM), or combination as analyzed by Western blotting. **E** Western blot analysis of MCL1 and MYC in Molm13-VR cells exposed to STM2457 at varing concentration for 48 h. **F** Schematic representation of the venetoclax-resistant primary AML-BMMNC in vitro study design. **G**, **H** The viability of primary AML cells treated with STM2457, venetoclax, or their combination at varying concentrations at 48 h by the CellTiter-Glo assay. Synergy was assessed by the SynergyFinder 3.0. **I** Protein levels of BCL2, MCL1, MYC, and PARP in primary AML cells treated with venetoclax(1 μM, 3 μM), STM2457(10 μM), or combination as analyzed by Western blotting. **J**, **K** Apoptosis assay of venetoclax-resistant primary AML cells treated with venetoclax(3 μM), STM2457(10 μM) or their combination at 48 h. The percentage of apoptotic cells after treatment was analyzed by flow cytometry. Percentages of apoptotic cells were calculated from three independent experiments. Results in the graphs are expressed as means ± SD. *p < 0.05, **p < 0.01, ***p < 0.001, ns not significant.

To further investigate whether STM2457 exerts anti-leukemic effects in venetoclax-resistant AML cells, we performed experiments with multiple venetoclax-resistant models. First, we constructed a Molm13 venetoclax-resistant (Molm13-VR) cell line by gradually increasing the dose of venetoclax (Fig. 6A). We found that the Drug Resistance Index (DRI) for Molm13 was over 30-fold as determined by cell viability assays. Notably, STM2457 showed comparable anti-proliferation potency in both Molm13-VR and parental cells (Fig. 6B). Consistent with previously reported venetoclax-resistant models [25, 30], we observed elevated levels of MCL1, BCL-XL, and MYC proteins in Molm13-VR cells, while BCL2 levels were decreased, and METTL3 levels were not changed significantly (Fig. 6C). Treatment with STM2457 and the combination effectively reduced MYC and MCL1 protein levels in this resistant model (Fig. 6D, E). In addition, in an artificially constructed MCL1-overexpressing MOLM13 cell line (oeMCL1-Molm13) (Supplementary Fig. S10A-B), which exhibited resistance to venetoclax (Supplementary Fig. S10C), STM2457 showed equivalent anti-proliferation potency (Supplementary Fig. S10D). Notably, silencing METTL3 or STM2457 treatment sensitized oeMCL1-Molm13 cells to venetoclax and restored the sensitivity (Supplementary Fig. S10E). The rescue efficiency was validated by Western blot assay in oeMCL1-Molm13 cells (Supplementary Fig. S10F). Therefore, based on these results, we confirmed that combination of venetoclax with STM2457 effectively mitigates the venetoclax resistance induced by MCL1 or MYC overexpression.

Finally, we collected bone marrow clinical specimens from patients with venetoclax-resistant relapses to evaluate the synergistic effect of STM2457 with venetoclax (Fig. 6F). The cell viability assay showed that STM2457 and venetoclax exhibited a strong synergistic effect in venetoclax-resistant BMMNCs (Fig. 6G, H). Meanwhile, we also observed that STM2457 enhanced the cytotoxic effect of venetoclax on drug-resistant AML cells by decreasing the protein levels of BCL2, MCL1 and MYC, while also inducing more apoptosis (Fig. 6I). Taken together, the combination of STM2457 and venetoclax may serve as a potential synergetic strategy for venetoclax-resistant AML therapy (Fig. 6J, K).

### Discovery of Isoliquiritigenin as a METTL3 inhibitor with potential anti-leukemia activity

**Fig. 7:**
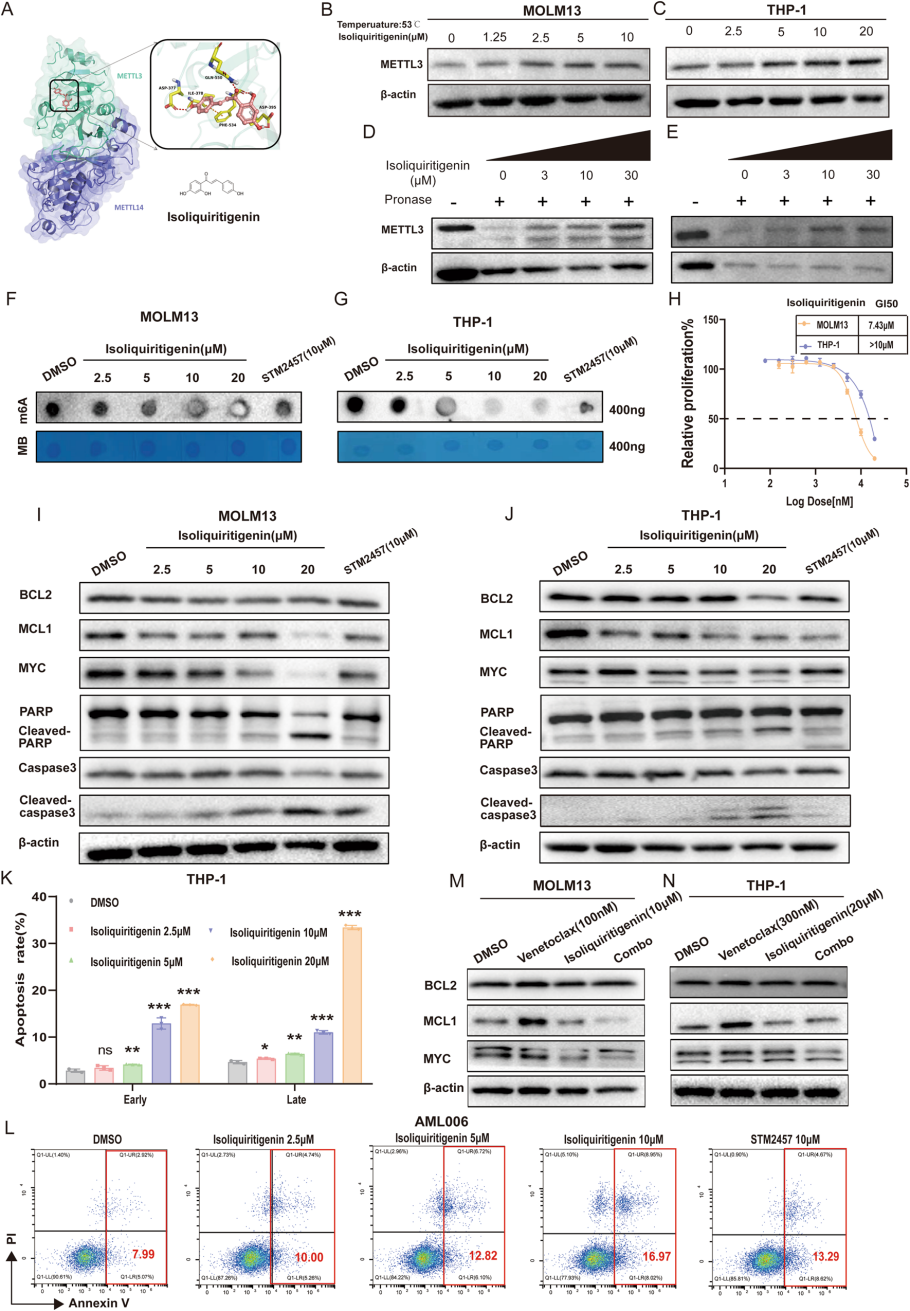
The natural product isoliquiritigenin targeting METTL3, shows potential anti- leukemia activity. **A** Molecular docking of isoliquiritigenin to the catalytic core of METTL3. **B**, **C** Cellular thermal shift assay (CETSA) analyses showed the stabilization of METTL3 in vitro with isoliquiritigenin of different concentrations treatment. **D**, **E** Drug affinity responsive target stability (DARTS) assays with Molm13 and THP-1 cell lysates in the presence of indicated concentration of isoliquiritigenin. **F**, **G** Dot blot analysis showed that isoliquiritigenin treatment reduced the N6-methyladenosine (m6A) abundance in Molm13 and THP-1 cells. **H** CellTiter-Glo assays in Molm13 and THP-1 cells after isoliquiritigenin treatment at 48 h. **I**, **J** Western blot analysis of isoliquiritigenin and STM2457 on the protein level of METTL3 targets and apoptosis in Molm13 and THP-1 cells. **K**, **L** Apoptosis assay of THP-1 and primary AML cells treated in varying concentrations of isoliquiritigenin for 48 h. The percentage of apoptotic cells after treatment was analyzed by flow cytometry. **M**, **N** Protein expression of BCL2, MCL1, and MYC in Molm13 and THP-1 cells treated with venetoclax(100 nM, 300 nM), isoliquiritigenin(10 μM, 20 μM), or combination as analyzed by Western blotting. Results in the graphs are expressed as means ± SD. *p < 0.05, **p < 0.01, ***p < 0.001, ns not significant.

Finally, we aimed to discover a novel METTL3 inhibitor with high potency. Through molecular docking and virtual screening of natural products library, we identified isoliquiritigenin as a compound that interacts with METTL3 in the MTase domain (Fig. 7A). The potential binding interaction between isoliquiritigenin and METTL3 was subsequently validated by the cellular thermal shift assay (CETSA) and drug affinity responsive target stability (DARTS) assays. These assays demonstrated that isoliquiritigenin conferred protection against high-temperature-induced aggregation and protease-mediated degradation, indicating that METTL3 is a potential target of isoliquiritigenin (Fig. 7B–E). In AML cells, isoliquiritigenin differentially reduced m6A modification levels (Fig. 7F, G). Additionally, cell viability assays were performed to assess the anti-proliferative ability of isoliquiritigenin in AML cells, with GI_50_ values of 7.43 μM for Molm13 cells and greater than 10 μM for THP-1 cells (Fig. 7H).

Isoliquiritigenin also mitigated the expression of target-related proteins (MCL1 and MYC) and apoptotic proteins (cleaved caspase-3 and cleaved PARP) in Molm13 and THP-1 cells in a dose-dependent manner (Fig. 7I, J). Flow cytometry analysis demonstrated isoliquiritigenin exhibited a concentration-dependent cytotoxic effect on THP-1 and primary venetoclax-resistant AML cells (Fig. 7K, L). In a subsequent study, the combination of isoliquiritigenin with venetoclax resulted in a more pronounced reduction in MCL1 and MYC protein expression (Fig. 7M, N). These results indicate that the novel METTL3 inhibitor, natural product isoliquiritigenin, exhibits potential anti-leukemia activity.

## Discussion

Although venetoclax has become extensively utilized in the clinical management of AML, relapse remains a frequent outcome with prolonged treatment [14, 15, 31]. Therefore, there is an ongoing effort to explore novel venetoclax combinations to enhance its anti-leukemic efficacy and to overcome venetoclax resistance. In this study, we propose a novel combinatory strategy wherein STM2457 synergistically enhances the anti-leukemic activity of venetoclax. We further investigate the underlying mechanisms of this synergy. Additionally, we demonstrate that STM2457 is effective in mitigating venetoclax resistance. Moreover, we identify isoliquiritigenin, a novel natural product, that targets METTL3 and exhibits promising anti-leukemic properties.

METTL3 functions as a pro-oncogene in AML, and targeting METTL3 has the potential to impede tumor progression [32]. STM2457 has demonstrated significant anti-tumor efficacy in both in vivo and in vitro leukemia models [19]. However, the combination of METTL3 inhibitors with venetoclax for AML treatment has not yet been investigated. Our study demonstrates that STM2457 can synergistically enhance the anti-tumor effects of venetoclax and improve the cytotoxic efficacy of venetoclax in both AML cell lines and primary AML specimens, with no significant toxicity towards healthy stromal HS-27A cells, BMMNCs, and PBMCs, thereby suggesting a favorable safety profile of this combination.

Resistance to venetoclax is primarily attributed to the upregulation of the MCL1 protein [24, 33–35]. The MYC oncogene is also associated with venetoclax resistance [25]. Our findings revealed that STM2457 reduced the expression of MCL1 and MYC. In addition, we validated the anti-leukemic efficacy of the combination with venetoclax in an in vivo AML model.

With regard to the effects of STM2457 on AML cells, Vu et al. have reported that MYC mRNA undergoes m6A modification by METTL3, which enhances its translation [18]. The inhibition of METTL3 by either knockdown or STM2457 treatment promotes the degradation of MYC by decreasing the levels of m6A-modified MYC mRNA, resulting in a reduction of MYC protein expression [19]. However, the specific effects of STM2457 on MCL1 protein levels remain to be investigated.

First, our enrichment analysis reveals that STM2457 primarily influences RNA and protein stability. Further, we demonstrated that STM2457 does not affect the stability of MCL1 mRNA, but promotes the degradation of MCL1 protein via the ubiquitin-proteasome pathway. Then, we integrated predictions from the UbiBrowser website with data from the comPPI database, which suggests that MCL1 may interact with the E3 ubiquitin ligase FBXW7. Current research has documented that in T-cell acute lymphoblastic leukemia, FBXW7 deletion results in the upregulation of MCL1, thereby conferring resistance to the BCL2 inhibitor ABT737 [36]. Our study corroborated these findings by demonstrating that STM2457 treatment enhances FBXW7 expression in AML cells, subsequently promoting the degradation of MCL1 protein. Additionally, FBXW7 knockdown mitigated the STM2457-induced degradation of MCL1 protein in AML cells. Collectively, these findings indicate that STM2457 facilitates MCL1 ubiquitination and subsequent protein degradation through the E3 ubiquitin ligase activity of FBXW7. And we observed the enhancement of FBXW7 mRNA stability in AML cells, along with an increase in FBXW7 mRNA and protein levels following the knockdown of METTL3.

**Fig. 8:**
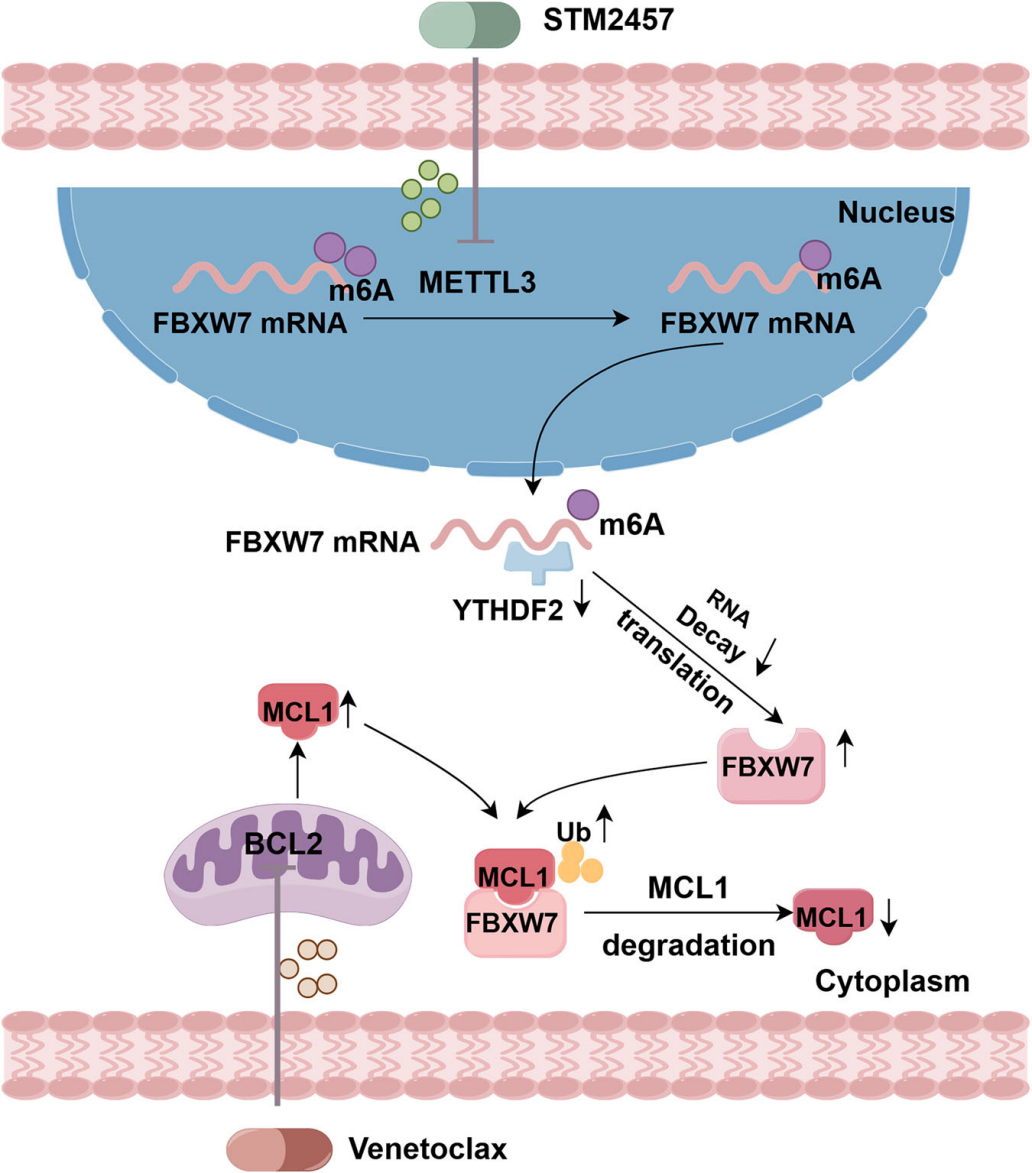
A proposed schematic model showing that STM2457 increases MCL1 ubiquitination via METTL3-YTHDF2-FBXW7 axis, which increases MCL1 protein degradation, revealing mechanisms to potentiate venetoclax anti-leukemic efficacy and mitigate venetoclax resistance.

Then, we identified YTHDF2 as the m6A reader for FBXW7 in AML. Taken together, in AML cells, METTL3 regulates FBXW7 expression in a YTHDF2-dependent manner. Inhibition of METTL3 by STM2457 enhances the mRNA stability of FBXW7, leading to elevated FBXW7 protein levels and subsequent MCL1 protein degradation (Fig. 8).

Additionally, the potential of STM2457 to combat resistance mechanisms was explored across various resistance models. Using venetoclax-induced resistant cells and artificially engineered MCL1-overexpressing cells, we validated that STM2457 effectively mitigated this resistance. Moreover, we confirmed the synergistic effect of STM2457 and venetoclax in primary leukemic cells that exhibit resistance to venetoclax. Together, our findings demonstrate that the combination of STM2457 and venetoclax effectively inhibits AML cell proliferation in vivo. Therefore, STM2457 may serve as a promising therapeutic option for ameliorating resistance to venetoclax in AML patients. In addition, natural products have a strong potential for drug development. Through molecular docking-based virtual screening, we identified isoliquiritigenin as a potential novel small molecule.

In conclusion, our study demonstrates that inhibition of METTL3 can synergize with venetoclax to inhibit AML cell proliferation and concurrently enhance the pro-apoptotic efficacy of venetoclax. Treatment with STM2457 led to the downregulation of MYC and MCL1, which further enhanced the activity of venetoclax. Specifically, STM2457 facilitated the ubiquitination of MCL1 and reduced its protein levels primarily through pharmaceutical targeting of METTL3, thereby enhancing the anti-leukemic potential of venetoclax and potentially attenuating resistance to venetoclax. Furthermore, we demonstrated the feasibility of co-administration in vivo and various venetoclax resistance models. Additionally, we identified the natural product isoliquiritigenin as a novel drug that potentially targets METTL3, which exhibits promising anti-leukemia properties.

## Supplementary information

SUPPLEMENTARY MATERIALS

Western blot original data

## Data Availability

All data for findings of this article will be shared on reasonable request to the corresponding author.
