# Supplementary material for: Targeting METTL3 mitigates venetoclax resistance via proteasome-mediated modulation of MCL1 in acute myeloid leukemia
Source: Cell Death Dis. 2025 Apr 1;16(1):233. doi: 10.1038/s41419-025-07560-w (PMC11962166; doi:10.1038/s41419-025-07560-w)
Supplement: Supplementary file 1 — SUPPLEMENTARY MATERIALS [file 41419_2025_7560_MOESM1_ESM.docx]

SUPPLEMENTARY MATERIALS

Targeting METTL3 Mitigates Venetoclax Resistance via Proteasome-Mediated Modulation of MCL1 in Acute Myeloid Leukemia

### Chang-qing Jiao^a,1^, Chen Hu^b,c,1^, Meng-hua Sun^a,1^, Yan Li^a^, Chao Wu^b,c^, Fei Xu^a^, Lei Zhang^a^, Fu-rong Huang^a^, Jun-jie Zhou^a^, Ji-fei Dai^a^, Min Ruan^a^, Wen-chao Wang^b,c,*^, Qing-song Liu^b,c,*^, Jian Ge^a,b,c,*^

^a^Department of Hematology, the First Affiliated Hospital of Anhui Medical University, Hefei 230022, China & Anhui Medical University, Hefei 230032, China

^b^Anhui Province Key Laboratory of Medical Physics and Technology, Institute of Health and Medical Technology, Hefei Institutes of Physical Science, Chinese Academy of Sciences, Hefei 230031, China

^c^Hefei Cancer Hospital, Chinese Academy of Sciences, Hefei 230031, China

^1^ These authors contributed equally to this work.

*Corresponding author

E-mail addresses: wwcbox@hmfl.ac.cn (Wen-chao Wang), qsliu97@hmfl.ac.cn (Qing-song Liu), [gejian@ahmu.edu.cn](mailto:gejian@ahmu.edu.cn) (Jian Ge)

**List of supplementary information**

1. Materials and Methods
2. Supplementary tables
3. Supplementary Figures
4. **Materials and Methods**

1.1 Compounds and antibodies

Compounds used in this study included: Venetoclax (Topscience, T2119), MG132 (Topscience, T2154), Isoliquiritigenin (Topscience, T0725), STM2457 (MedChemExpress, HY-134836), and cyclohexane (MedChemExpress, HY-12320).

Antibodies used in this study included: anti-BCL2 (Cell signaling, #4223), anti-PARP (Cell signaling, #9542), anti-cleaved caspase3 (Cell signaling, #9661), anti-caspase3 (Cell signaling, #14220), ubiquitin(Cell signaling, #3936), normal Rabbit IgG(Cell signaling, #2729), anti-YTHDF2 (abcam,#ab220163), anti-m6A (Abcam, #ab208577), anti-Ki67 (Abcam, #ab15580),anti-MCL1(cell signaling, #94296), anti-MCL1(Proteintech, #16225-1-AP), anti-MYC(Proteintech, #10828-1-AP), anti-FBXW7(Proteintech, #55290-1-AP), anti-BCL-XL(Proteintech, #66020-1-Ig), APC anti-Human CD45 (Proteintech, #APC-65109),recombinant anti-beta Actin(Servicebio, #ZB 15001-HRP-100), FLAG(Sigma-Aldrich, #F1804).

1.2 Cell culture

Molm13, Molm14, HL-60, U937, OCI-AML-3, THP-1, EOL-1, Kasumi-1, NOMO-1, HEL, NB4, and HS-27A cells were cultured in RPMI1640 medium with 10% FBS and 1% penicillin/streptomycin (KeyGEN). THP-1 cells were cultured in RPMI1640 medium with 10% FBS, 0.05mM β-mercaptoethanol, and 1% penicillin/streptomycin. OCI-AML-3 cells were cultured with α-MEM media (Corning) with 10% FBS and 1% penicillin/streptomycin. HEK-293T cells were cultured in DMEM (KeyGEN) with 10%FBS and 1% penicillin/streptomycin. Cell lines used in this study were cultured with 5% CO2 at 37°C.

1.3 Cell proliferation, apoptosis and cell cycle

Cell Proliferation and apoptosis study were carried out as previously study described[1]. In short, CellCounting-Lite 2.0 (Vazyme) was added to evaluate the cell viability. The cell apoptosis was detected by Annexin V-FITC/PI double staining(Vazyme). After staining by annexin V-FITC and PI staining solution, and subjected to flow cytometry (CytoFLEX, BECKMAN).

1.4 Mitochondrial membrane potential (MMP) assay

MMP changes were evaluated using the Enhanced MMP assay kit with JC-1 (Beyotime). AML cells were incubated with JC-1 for 20 minutes at 37°C, washed twice, and imaged with a fluorescence microscope. Red fluorescence indicates healthy mitochondria, while green fluorescence indicates MMP loss.

1.5 EdU assay

EdU incorporation assay Cell proliferation was detected according to manufacturer’s instructions(Beyotime). Briefly, cells were incubated with 50 mM EdU for 4h at 37C. After EdU staining, cells were stained with Hoechst 33342.

1.6 Plasmid construction

PCR reactions were performed using the PrimeSTAR Max DNA Polymerase (TAKARA). All primer sequences used are listed in Supplementary Table S5. To generate OE-METTL3, the fragments of truncated METTL3 gene was amplified by PCR and insert into pLVX-Puro vector (clontech) using T4 DNA ligase (TAKARA). To generate shMETTL3 plasmids, the shMETTL3#1 and shMTL3#2 sequences were synthesized by general bio, annealed oligos were cloned into the pLVX-shRNA(clontech) vector. pLV3-CMV-FBXW7(human)-3xFLAG-Puro was purchased from MIAOLING BIOLOGY. All constructs were checked by Sanger sequencing.

1.7 Cell transfection and lentivirus infection

For transient transfection, cells were transfected using CALNPTM RNAi in vitro(D-nano Therapeutics) for siRNA(general biol). The cells cultured for 48h with medium, then total RNA and protein were extracted for analysis. Selected sequences are listed in Table S6. Further, pLV3-CMV-FBXW7(human)-3xFLAG-Puro plasmid was transfected into HEK293T cells for 48h. For lentivirus production, related plasmids were cotransfected with packaging vectors psPAX2 and pMD2.G into HEK293T cells using LipoFiter(HANBIO). Infectious lentivirus particles were harvested at 48h after transfection. The lentivirus of overexpressing MCL1 gene were purchased from GenePharma Co., Ltd.

1.8 Western blot

Western blot was carried out as previously study described[1].

1.9 CO-immunoprecipitation(Co-IP)

Harvested cells were cleaned twice with PBS, sonicated in IP buffer (Beyotime), then centrifuged at 13000 rpm at 4℃ for 30 min. The levels of identified proteins were examined using the indicated antibodies to normalize the input. Briefly, clarified lysates were incubated with primary antibodies or an isotype-matched negative control IgG. Thereafter, the sample-antibody mixtures were rotated together with Protein G Beads (MedChemExpress) overnight at 4℃, cleaned three times with IP Lysis Buffer and gathered by magnetic separation. After boiling in 2×SDS-PAGE (Beyotime), proteins were subjected to western blot analysis.

1.10 Protein ubiquitination and stability assays

In brief, cells were pre-treated with MG132 for 6h before being harvested. Then, the expression level and ubiquitination status of MCL1 protein were determined by WB. To evaluate protein stability, cells were pre-treated with CHX for the indicated times.

1.11 Molm13 xenograft tumor model

All animals were housed in a specific pathogen-free facility and used according to the animal care regulations of Anhui Medical University. The serial number of the ethical approval for animal experiments is LLSC20232152. Mouse xenograft tumor model was carried out as previously described[1]. Briefly, 5million Molm13 cells in RPMI-1640 medium were injected into the subcutaneous space on the right flank of NOD-SCID mice. Animals were randomized into groups with 5 mice in each for efficacy studies. STM2457 (50 mg/kg) was administered daily via i.p., and venetoclax (100 mg/kg) via p.o. Body weight and tumor growth were monitored twice daily. Tumor volume (mm³) was calculated using the formula: (W²× L) / 2, where width (W) is the smaller measurement and length (L) is the larger measurement.

1.12 Molm13 engraft tumor model

Five-week old female NCG mice were purchased from GemPharmatech (Nanjing, China). All animals were housed in a specific pathogen free facility and used according to the animal care regulations of Anhui Medical University. NCG mice were intraperitoneal injection cyclophosphamide (CTX) 50 mg/kg daily for two days. 5 million Molm13 cells in 0.3 mL 1640 medium were injected by intravenous after 24 h. Venetoclax(50mg/kg) or STM2457(50mg/kg) were administered daily by p.o. or i.p. . Mice were monitored daily and were euthanized when moribund or at early signs of hind limb paralysis.

1.13 HE staining and immunohistochemistry

Immunohistochemistry(IHC) and hematoxylin & eosin (H&E) staining Formalin-fxed parafn-embedded sections underwent H&E staining or were subjected to immunostaining using anti-ki67 and tunel , following a standard IHC protocol.

1.14 Molecular docking

The crystal structure of METTL3 was obtained from the Protein Data Bank (PDB ID: 5K7U). Water molecules and the original ligand were manually removed by using PyMol software(version 1.8). Prediction of the binding pose of isoliquiritigenin(provided by Topscience) was carried out by Autodock (version 4.2.6)[2] Prepare_ligand4.py and prepare_recptor4.py scripts from AutoDockTools 1.5.6 were used to prepare the initial files including adding charges and hydrogen atoms. A grid box of 60 × 60 × 60 with a spacing of 0.375Å was then set to enclose the whole binding site. The Lamarckian genetic algorithm (LGA) was adopted to search the optimal binding poses. The specific docking settings were as follows: trials of 100 dockings, 300 individuals per population with a crossover rate of 0.8 and the local search rate was set to 0.06. Other parameters were set as defaults during the docking.

1.15 The Cellular Thermal Shift Assay (CETSA) and Drug affinity responsive target stability (DARTS)

CETSA study were carried out as previously study described[3].

DARTS: Cells were lysed with NP-40, and cell lysates were aliquoted into different tubes and incubated with drug at different concentrations at 4 ℃ for 4 h, followed by pronase treatment at room temperature for 10min. Subsequently, the reactions were terminated by adding 2xSDS loading buffer, and thesamples were analyzed by immunoblotting.

1. **Supplementary tables**

Tables S1 The detailed information of samples we utilized in the study.

| Patient | Gender | Age | Disease status | FAB | Blast purity(%) |
| --- | --- | --- | --- | --- | --- |
| AML003 | Female | 56 | Newly-diagnosed | M5 | 79% |
| AML007 | Male | 73 | Newly-diagnosed | M5 | 93% |
| AML004 | Female | 49 | R/R AML | M4 | 84% |
| AML005 | Female | 54 | R/R AML | M4 | 71% |
| AML006 | Male | 68 | R/R AML | M5 | 81% |
| Donor | Male | 28 | Healthy |  |  |
| Donor | Female | 32 | Healthy |  |  |

Tables S2 Primer pairs used for the RT-qPCR

| mRNA | Forward primer(5'-3') | Reverse primer(5'-3') |
| --- | --- | --- |
| METTL3 | TTGTCTCCAACCTTCCGTAGT | CCAGATCAGAGAGGTGGTGTAG |
| BCL2 | TCATGTGTGTGGAGAGCGTC | TCCACAAAGGCATCCCAGC |
| MCL1 | GGGCAGGATTGTGACTCTCATT | GATGCACGCTTTCTTGGTTTATGG |
| c-Myc | CCTGGTGCTCCATGAGGAGAC | CAGACTCTGACCTTTTGCCAGG |
| β-actin | CCTGGCACCCAGCACAAT | GGGCCGGACTCGTCATAC |
| YTHDF2 | CCAAAAGGTCAAGGAAACAAA | GGAAAAGCCAATGGAGGG |
| HUWE1 | TTGGACCGCTTCGATGGAATA | TGAAGTTCAACACAGCCAAGAG |
| TRCP | CCAGACTCTGCTTAAACCAAGAA | GGGCACAATCATACTGGAAGTG |
| FBXW7 | GGCCAAAATGATTCCCAGCAA | ACTGGAGTTCGTGACACTGTTA |

Table S3 Synergy scores matrix of Molm13

| **Drug1** | **Drug2** | **Conc1** | **Conc2** | **Relative_inhibition** | **Synergy** | **concUnit** |
| --- | --- | --- | --- | --- | --- | --- |
| STM2457 | Venetoclax | 0 | 0 | 0 | 0 | nM |
| STM2457 | Venetoclax | 1000 | 0 | 7.486475567 | 0 | nM |
| STM2457 | Venetoclax | 3000 | 0 | 20.70297143 | 0 | nM |
| STM2457 | Venetoclax | 10000 | 0 | 66.81941867 | 0 | nM |
| STM2457 | Venetoclax | 0 | 3 | 1.2476811 | 0 | nM |
| STM2457 | Venetoclax | 1000 | 3 | 9.374264767 | 1.929327594 | nM |
| STM2457 | Venetoclax | 3000 | 3 | 34.88807927 | 11.01526599 | nM |
| STM2457 | Venetoclax | 10000 | 3 | 70.4266855 | 3.77400276 | nM |
| STM2457 | Venetoclax | 0 | 10 | 8.646396 | 0 | nM |
| STM2457 | Venetoclax | 1000 | 10 | 20.61949343 | 9.458244715 | nM |
| STM2457 | Venetoclax | 3000 | 10 | 38.19550463 | 8.446500361 | nM |
| STM2457 | Venetoclax | 10000 | 10 | 75.45741807 | 7.501567623 | nM |
| STM2457 | Venetoclax | 0 | 30 | 14.5453665 | 0 | nM |
| STM2457 | Venetoclax | 1000 | 30 | 34.84027993 | 17.53902768 | nM |
| STM2457 | Venetoclax | 3000 | 30 | 55.21470143 | 17.22086043 | nM |
| STM2457 | Venetoclax | 10000 | 30 | 80.69739977 | 10.84131576 | nM |
| STM2457 | Venetoclax | 0 | 100 | 28.66730693 | 0 | nM |
| STM2457 | Venetoclax | 1000 | 100 | 44.46806537 | 10.57251077 | nM |
| STM2457 | Venetoclax | 3000 | 100 | 68.95031583 | 21.17779277 | nM |
| STM2457 | Venetoclax | 10000 | 100 | 89.73863143 | 15.17152944 | nM |
| STM2457 | Venetoclax | 0 | 300 | 42.80022567 | 0 | nM |
| STM2457 | Venetoclax | 1000 | 300 | 55.02223887 | 7.249700846 | nM |
| STM2457 | Venetoclax | 3000 | 300 | 73.07823677 | 19.31324595 | nM |
| STM2457 | Venetoclax | 10000 | 300 | 95.73094887 | 16.30791407 | nM |
| STM2457 | Venetoclax | 0 | 1000 | 58.54107413 | 0 | nM |
| STM2457 | Venetoclax | 1000 | 1000 | 74.11987853 | 14.90641851 | nM |
| STM2457 | Venetoclax | 3000 | 1000 | 85.8341922 | 21.16452815 | nM |
| STM2457 | Venetoclax | 10000 | 1000 | 98.89977437 | 13.90631736 | nM |

Table S4 Synergy scores matrix of THP-1

| **Drug1** | **Drug2** | **Conc1** | **Conc2** | **Relative_inhibition** | **Synergy** | **concUnit** |
| --- | --- | --- | --- | --- | --- | --- |
| STM2457 | Venetoclax | 0 | 0 | 0 | 0 | nM |
| STM2457 | Venetoclax | 3000 | 0 | 15.13308483 | 0 | nM |
| STM2457 | Venetoclax | 10000 | 0 | 34.9484142 | 0 | nM |
| STM2457 | Venetoclax | 30000 | 0 | 40.89671803 | 0 | nM |
| STM2457 | Venetoclax | 0 | 30 | 0.597972733 | 0 | nM |
| STM2457 | Venetoclax | 3000 | 30 | 22.82649187 | -3.741172787 | nM |
| STM2457 | Venetoclax | 10000 | 30 | 44.27999203 | 10.10994705 | nM |
| STM2457 | Venetoclax | 30000 | 30 | 58.12789033 | 18.66846558 | nM |
| STM2457 | Venetoclax | 0 | 100 | 11.8691665 | 0 | nM |
| STM2457 | Venetoclax | 3000 | 100 | 37.7146226 | 10.5180289 | nM |
| STM2457 | Venetoclax | 10000 | 100 | 53.56548267 | 16.98580655 | nM |
| STM2457 | Venetoclax | 30000 | 100 | 68.85401247 | 27.39628169 | nM |
| STM2457 | Venetoclax | 0 | 300 | 16.08595537 | 0 | nM |
| STM2457 | Venetoclax | 3000 | 300 | 48.38624963 | 22.07926194 | nM |
| STM2457 | Venetoclax | 10000 | 300 | 76.0429553 | 38.11839044 | nM |
| STM2457 | Venetoclax | 30000 | 300 | 78.88059623 | 35.33383511 | nM |
| STM2457 | Venetoclax | 0 | 1000 | 33.8102802 | 0 | nM |
| STM2457 | Venetoclax | 3000 | 1000 | 58.30275657 | 20.75977253 | nM |
| STM2457 | Venetoclax | 10000 | 1000 | 78.0362643 | 26.74430849 | nM |
| STM2457 | Venetoclax | 30000 | 1000 | 86.14684977 | 30.91004151 | nM |
| STM2457 | Venetoclax | 0 | 3000 | 47.39184443 | 0 | nM |
| STM2457 | Venetoclax | 3000 | 3000 | 67.42199763 | 17.11035641 | nM |
| STM2457 | Venetoclax | 10000 | 3000 | 88.2538269 | 27.44374794 | nM |
| STM2457 | Venetoclax | 30000 | 3000 | 94.25683667 | 30.27437411 | nM |

Table S5 Oligo sequences uesed for PCR

| shMETTL3-1 | GCTACCTGGACGTCAGTATCT |
| --- | --- |
| shMETTL3-2 | CGCTCAACATACCCGTACTACA |
| oeMETTL3 | F:ATGTTTGGCCTCAAAAGAAACGCGGT |
|  | R:CTATCTTATTAGATATGCCAAACCAGCTCCTACTCCAGCA |

Table S6 The siRNA oligo sequences used for the cell transfection

| Targets | Sense (5’-3’) | Anti-sense (5’-3’) |
| --- | --- | --- |
| si-NC | UUCUCCGAACGUGUCACGUTT | ACGUGACACGUUCGGAGAATT |
| si1-METTL3 | CUACAGAUCCUGAGUUAGATT | UCUAACUCAGGAUCUGUAGTT |
| si2-METTL3 | GUUGAUCUGGAGAUAGAGATT | UCUCUAUCUCCAGAUCAACTT |
| si1-FBXW7 | GGUUCCUGUUGAUCUUAAATT | UUUAAGAUCAACAGGAACCTT |
| si2-FBXW7 | GGAUCUCUUGAUACAUCAATT | UUGAUGUAUCAAGAGAUCCTT |
| si3-FBXW7 | GGAGUAUGGUCAUCACAAATT | UUUGUGAUGACCAUACUCCTT |
| si1-YTHDF2 | AGAAUAAACCAGUGACCAATT | UUGGUCACUGGUUUAUUCUTT |
| si2-YTHDF2 | GGUGGAUGGUAAUGGAGUATT | UACUCCAUUACCAUCCACCTT |
| si3-YTHDF2 | GCACAGAGCAUGGUAACAATT | UUGUUACCAUGCUCUGUGCTT |

**3.Supplementary Figures**


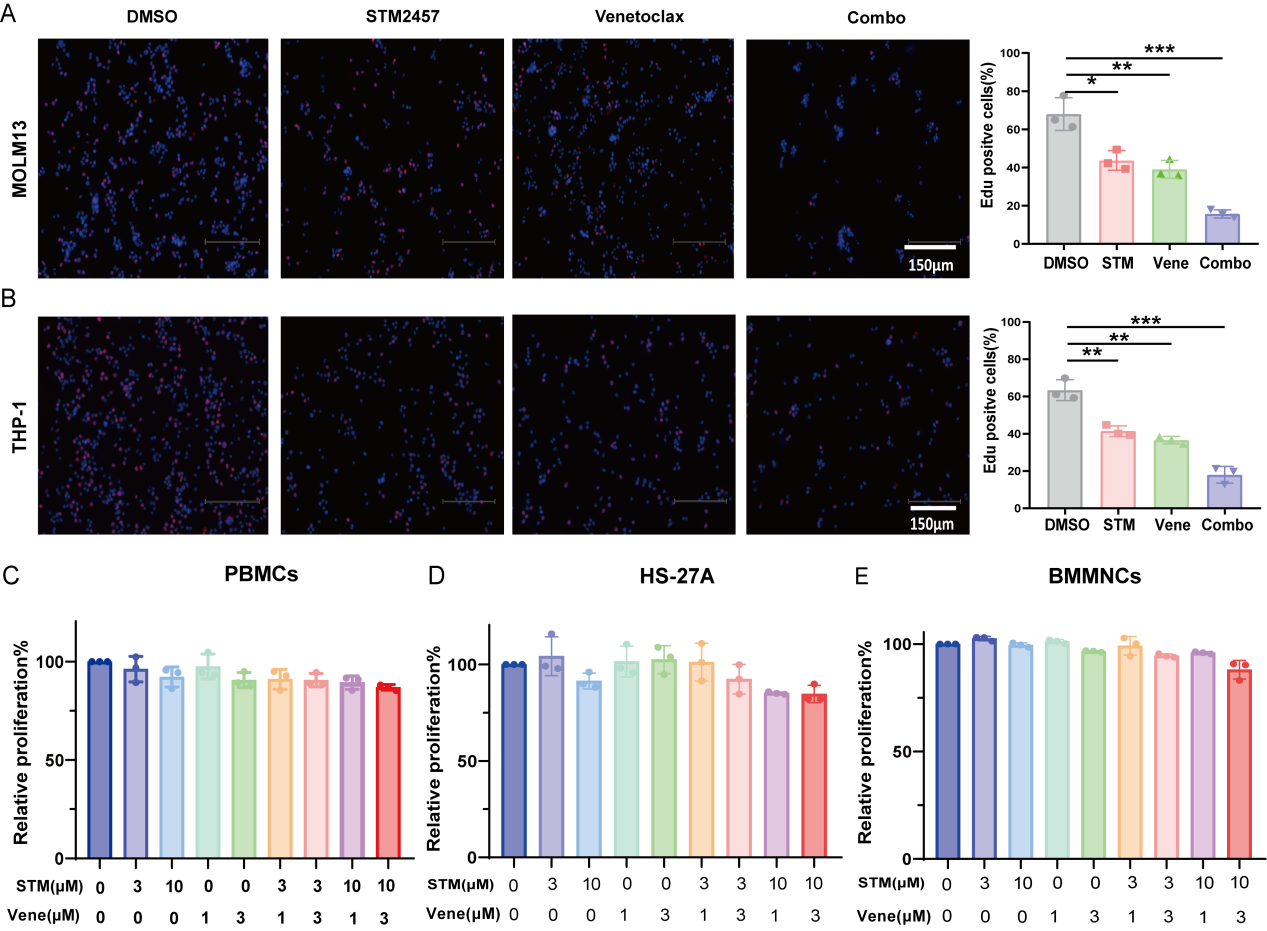


Figure S1 The antiproliferative effect of STM2457 combined with venetoclax on AML cells and the toxicity to normal hematopoietic cells.

A,B) EdU incorporation assay of Molm13 or THP- 1 cells treated with drugs for 48h. Scale bars: 150μm.

C-E) PBMCs, HS-27A, and BMMNCs were cultured in the presence of venetoclax and STM2457 alone or combination for 48 h. Cell viability was measured by CellTiter-Glo assay. Data were expressed ± SEM. All by Student’s t-test. *p<0.05; **p<0.01;***p<0.001;ns,not significant.


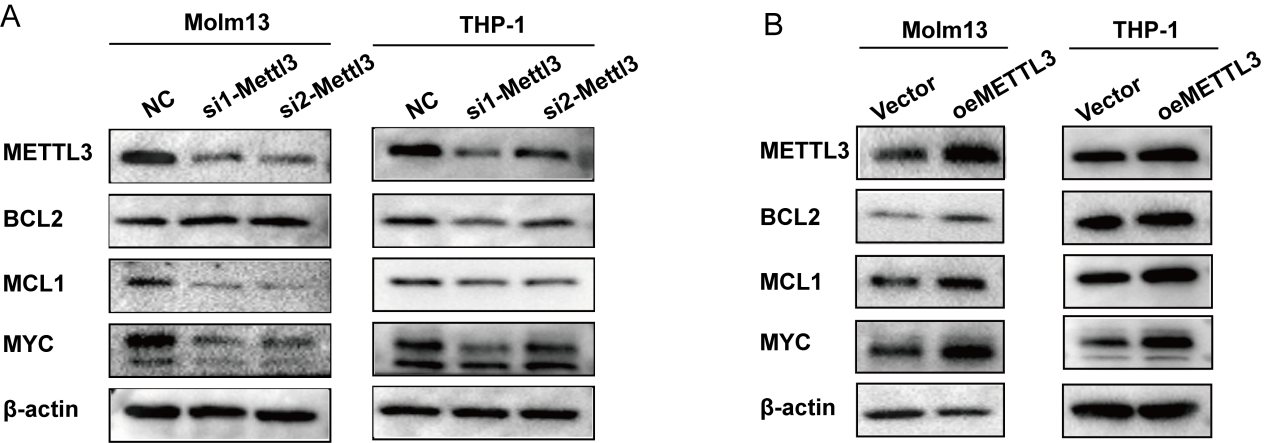


Figure S2 Targeting METTL3 in Molm13 and THP-1 cells affect MCL1 and MYC expression.

A) Western blot analysis of METTL3, BCL2, MCL1, and MYC protein expression by silencing METTL3 in Molm13 and THP-1 cells.

B) Western blot analysis of METTL3, BCL2, MCL1, and MYC protein expression by overexpression METTL3 in Molm13 and THP-1 cells.


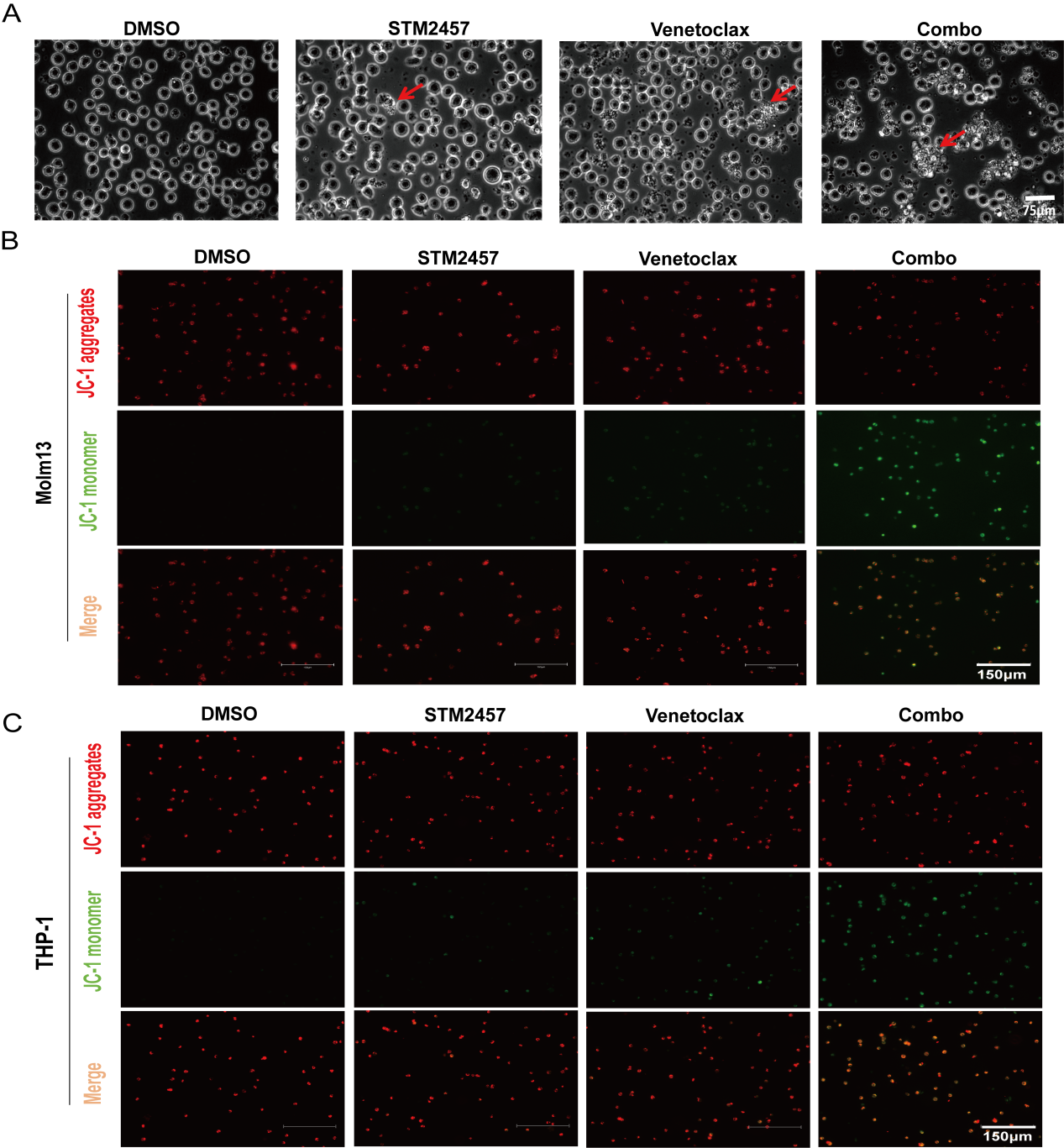


Figure S3 STM2457 enhances the apoptosis of AML cells induced by venetoclax.

1. Representative light microscopy images of the same treated with drugs for 48h. The red arrowheads indicated the characteristic balloons on the cell membrane. Scale bars: 75μm.

B, C) Mitochondrial membrane potential was detected with JC-1 staining after treatment with venetoclax(100nM, 300nM), STM2457(3μM, 10μM), or their combination for 48 h. Red fluorescence (JC-1 aggregate form) represents normal membrane potential, and green fluorescence (JC-1 monomer form) represents mitochondrial membrane potential depolarization. Scale bars: 150μm.

###
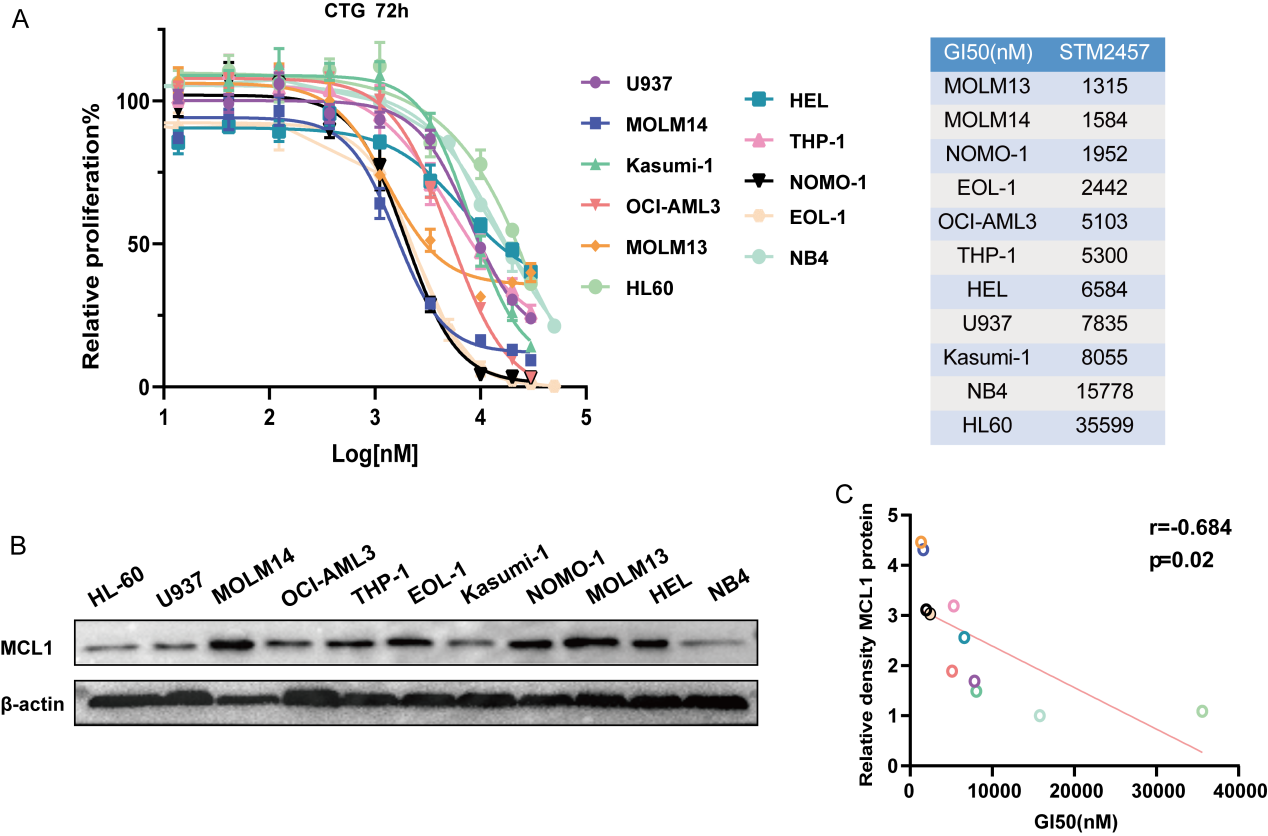


Figure S4 The GI_50_ values of STM2457 was significant negative correlation with MCL protein level.

A) The GI_50_ values of STM2457 to different AML cell lines were measured by CellTilter-Glo assay for 72h.

B) Protein expression of MCL1 in different cell lines as analyzed by western blotting.

C) The correlation plot of baseline MCL1 protein levels and GI_50_ values for different AML cell lines.


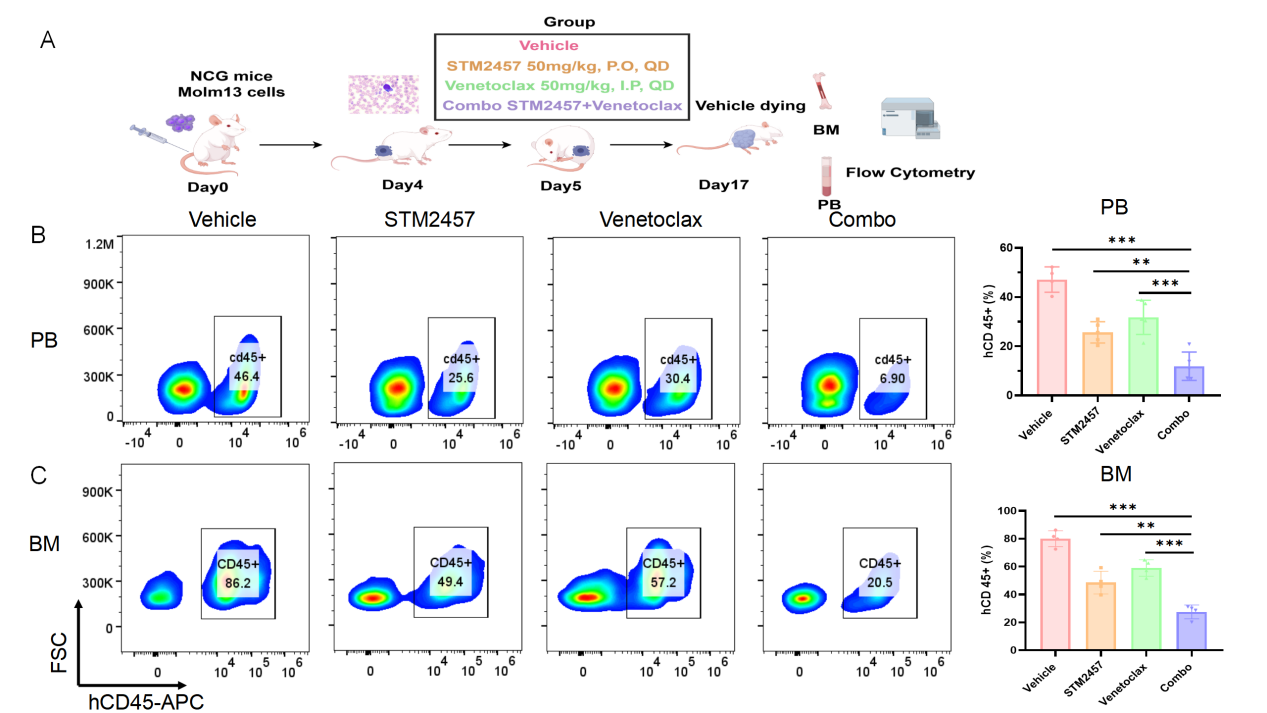


Figure S5 STM2457 enhances the anti-leukemic effect of venetoclax in Molm13 engrafment model. A) Schematic representation of the AML cells in vivo study design.

B, C) Flow cytometry analysis of human CD45+ leukemia cell in PBMC and bone marrow. Results in the graphs are expressed as means±SD. *p < 0.05, **p < 0.01, ***p < 0.001, ns, not significant


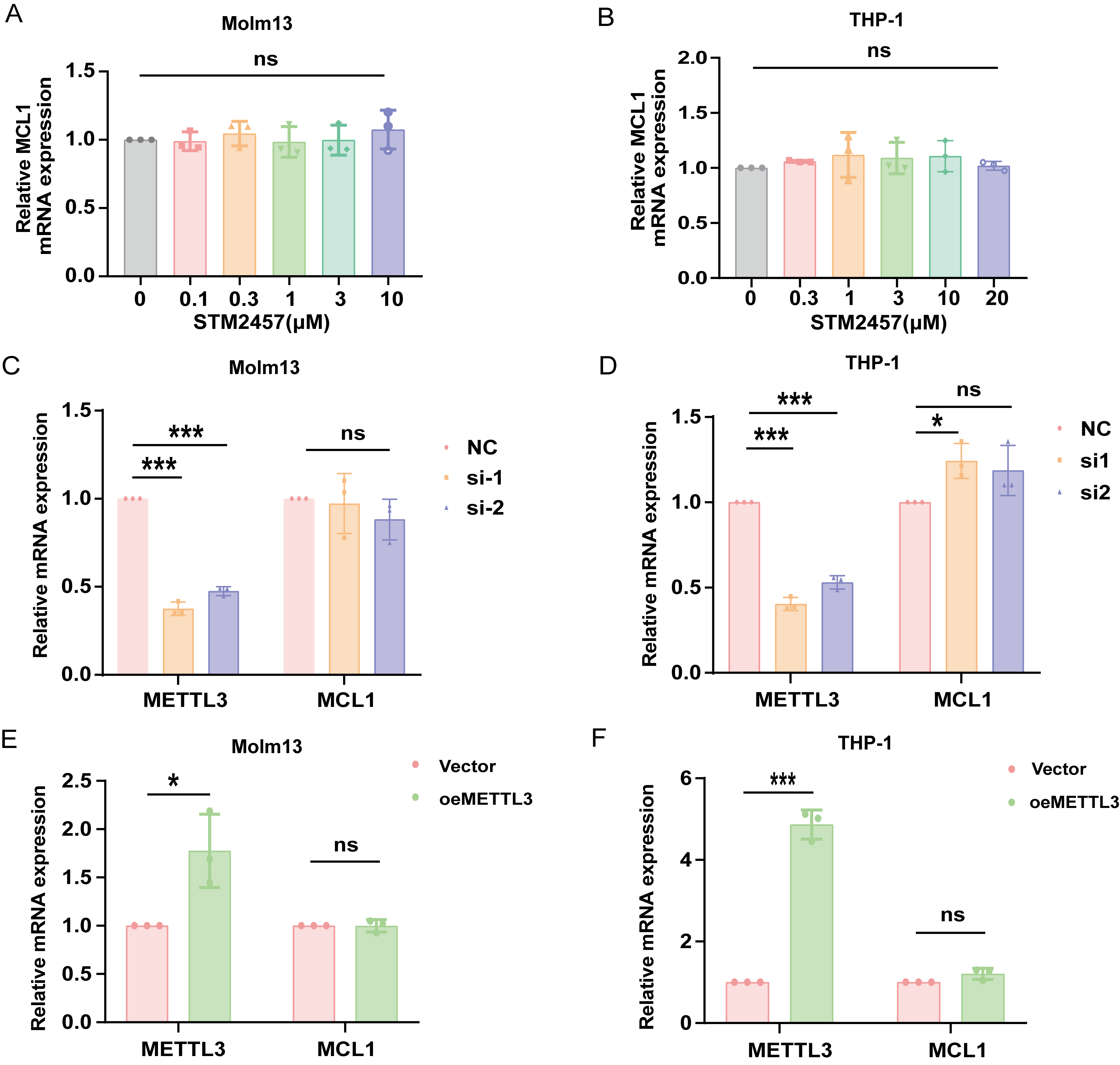


Figure S6 Targeting METTL3 in Molm13 and THP-1 cells do not affect MCL1 mRNA transcript levels.

A, B) Transcriptional levels of MCL1 to Molm13 and THP-1 cells treated with STM2457 at varing concentration for 48h.

C, D) RT-qPCR analysis of METTL3 and MCL1 mRNA levels by silencing METTL3 in Molm13 and THP-1 cells.

E, F) RT-qPCR analysis of METTL3 and MCL1 mRNA levels by overexpression METTL3 in Molm13 and THP-1 cells.

###
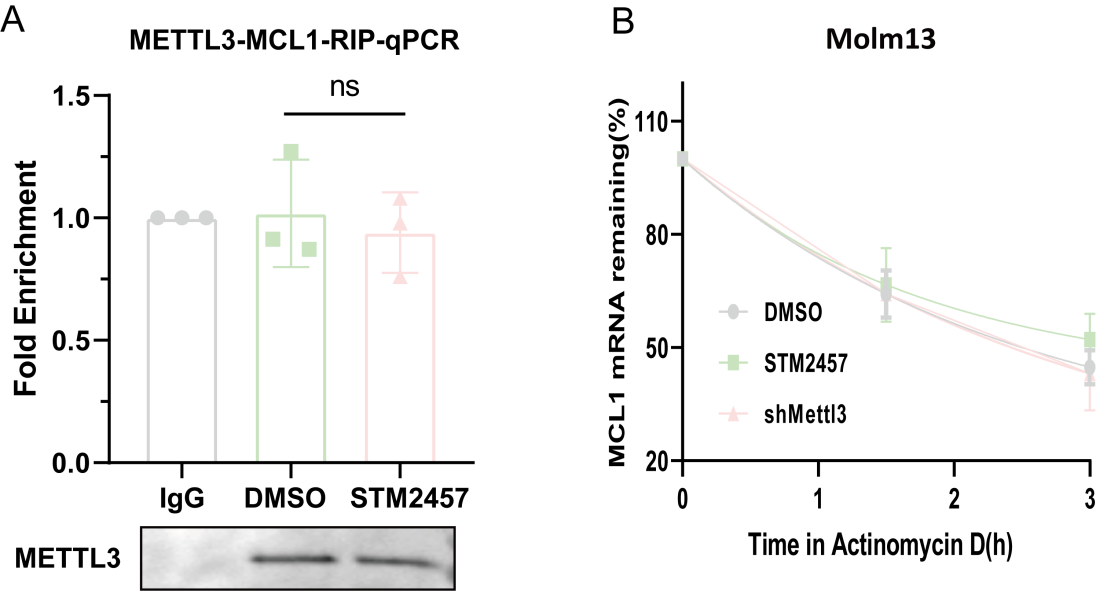


Figure S7 A) The interaction between METTL3 and MCL1 mRNA analyzed by RIP-qPCR assay.

B) Effects of sh-METTL3 or STM2457 on the mRNA level of MCL1 in Molm13 cells. Cells were treated with Actinomycin D(5μg/ml) at indicated times.


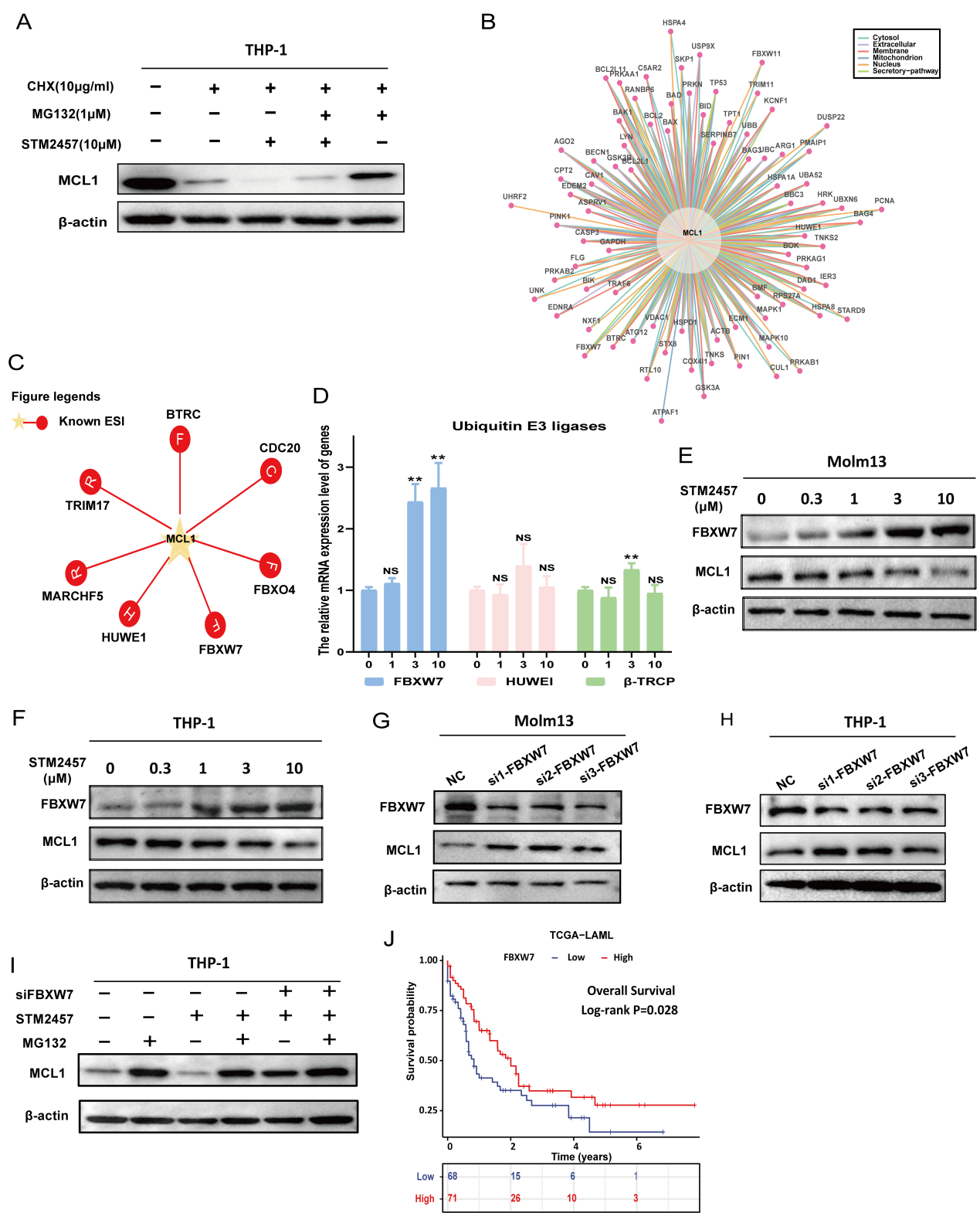


Figure S8 STM2457 degrades MCL1 protein via E3 ubiquitin ligase FBXW7.

1. Effects of MG132 treatment on MCL1 expression in THP-1 cells. THP-1 cells pretreated with or without CHX(10μg/ml), MG132(1μM), and STM2457(10μM) for 1.5h.
2. The proteins that may potentially interact with MCL1 have been identified by ComPPI(https://comppi.linkgroup.hu/).
3. The E3 ubiquitin ligase of MCL1 was predicted using the UbiBrowser website(http://ubibrowser.bio-it.cn/ubibrowser_v3/).
4. RT-qPCR analysis of FBXW7, HUWE1, and BTRC mRNA in Molm13 with varying dose of STM2457 for 48h.

E, F) Western blot analysis of the protein levels of FBXW7 and MCL1 on Molm13 and THP-1 cells treated with STM2457 at varying concentrations for 48h.

G, H) Western blot analysis of FBXW7 and MCL1 by siRNA FBXW7 mRNA in Molm13 cells and THP-1 cells.

1. After FBXW7 silencing and STM2457 treatment, Molm13 cells were co-cultured with or without MG132 (1μM) for 4 hours, and MCL1 protein expression was assessed by western blotting.
2. Kaplan-Meier analysis of OS curves for AML patients with FBXW7-low and FBXW7-high expression form TCGA-LAML dataset.


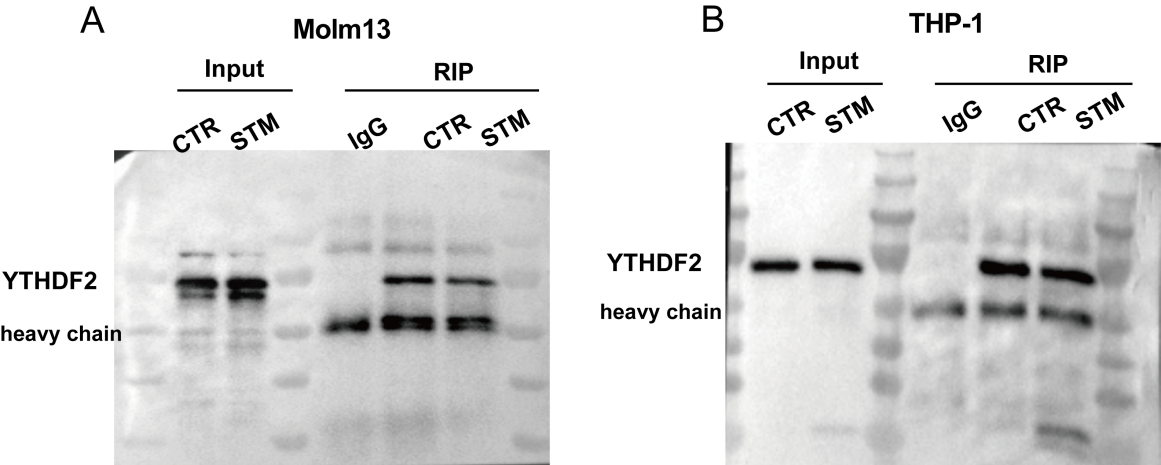


Figure S9 Anti-YTHDF2 RIP test the interaction between FBXW7 mRNA and YTHDF2 in Molm13 and THP-1 cells. Molm13 and THP-1 cells were treated with STM2457(10μM) for 48h, then Anti-YTHDF2 and Ig-G antibody were used for RIP assay.


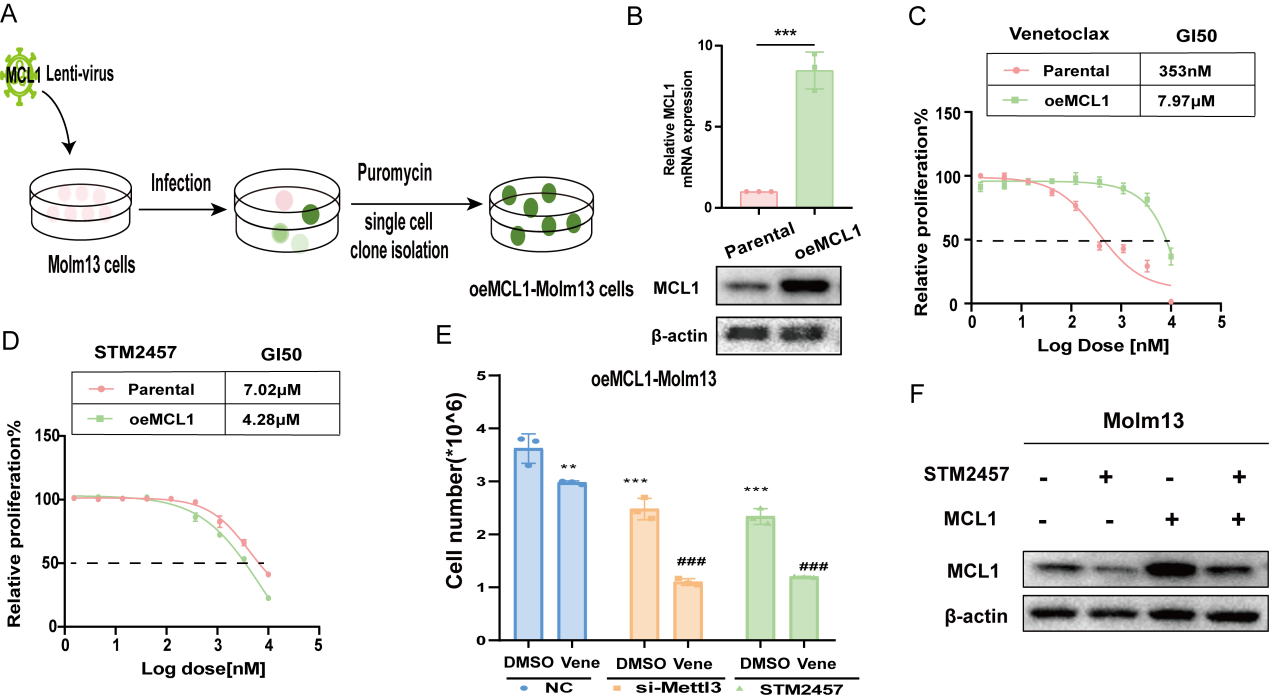


Figure S10 STM2457 Mitigates venetoclax resistance in Molm13 cells overexpressing MCL1.

A）Schematic illustration for constructing overexpressing MCL1 Molm13 cells.

B) Validating oe-MCL1-Molm13 cells by western blotting and RT-qPCR assay. C,D) CellTiter-Glo assay for Molm13 and oeMCL-Molm13 cells treated with venetoclax and STM2457.

E) Targeting METTL3(si-METTL3 or STM2457), oeMCL1 molm13 cells for cell viability assay of Venetoclax. * Comparing with NC-DMSO is significant, # Comparing with NC-Vene is significant.

1. The rescue efficiency was validated by western blotting assay in oe-MCL1 Molm13 cells.

Results in the graphs are expressed as means±SD. *p < 0.05, **p < 0.01, ***p < 0.001, ###p < 0.001, ns, not significant

[1] C. Hu, L. Shen, F. Zou, Y. Wu, B. Wang, A. Wang, C. Wu, L. Wang, J. Liu, W. Wang, Q. Liu, Predicting and overcoming resistance to CDK9 inhibitors for cancer therapy, Acta Pharm Sin B, 13 (2023) 3694-3707.

[2] G.M. Morris, R. Huey, W. Lindstrom, M.F. Sanner, R.K. Belew, D.S. Goodsell, A.J. Olson, AutoDock4 and AutoDockTools4: Automated docking with selective receptor flexibility, J Comput Chem, 30 (2009) 2785-2791.

[3] H. Mei, H. Wu, J. Yang, B. Zhou, A. Wang, C. Hu, S. Qi, Z. Jiang, F. Zou, B. Wang, F. Liu, Y. Chen, W. Wang, J. Liu, Q. Liu, Discovery of IHMT-337 as a potent irreversible EZH2 inhibitor targeting CDK4 transcription for malignancies, Signal Transduct Target Ther, 8 (2023) 18.
