# Supplementary material for: Targeting METTL3 mitigates venetoclax resistance via proteasome-mediated modulation of MCL1 in acute myeloid leukemia
Source: Cell Death Dis. 2025 Apr 1;16(1):233. doi: 10.1038/s41419-025-07560-w (PMC11962166; doi:10.1038/s41419-025-07560-w)

Fig 2A MOLM13

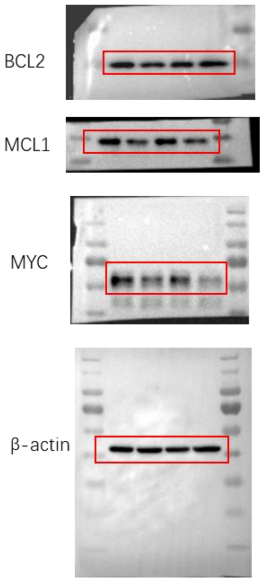

Fig 2B THP-1

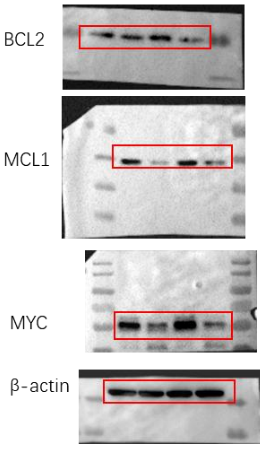

Fig 2C MOLM13

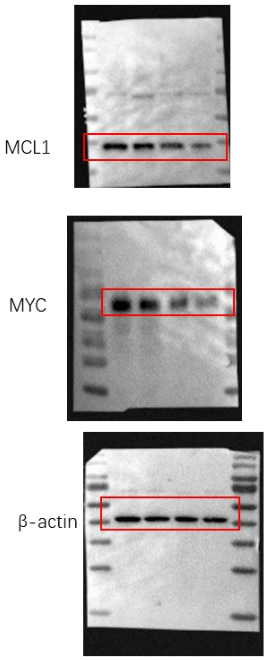

Fig 2D THP-1

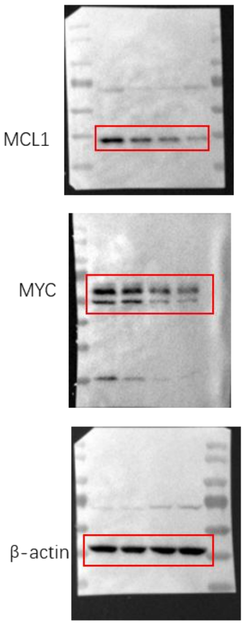

Fig 2E

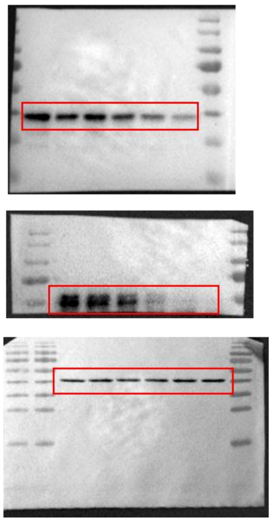

Fig 2F

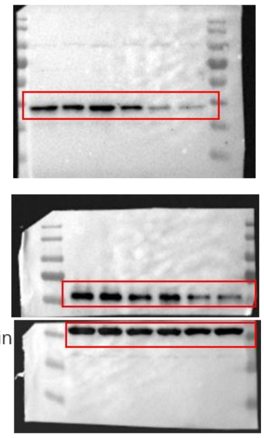

Fig 2H

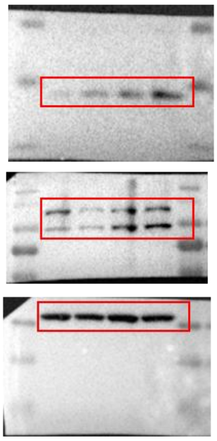

Fig 2I

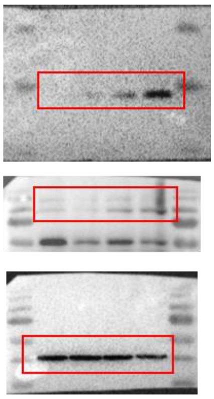

Fig 3E

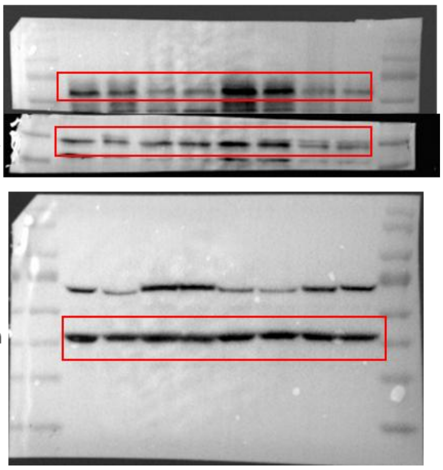

Fig 4B MOLM13

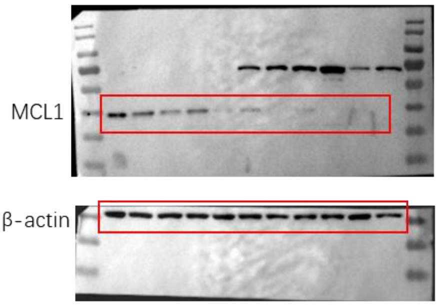

Fig 4C THP-1

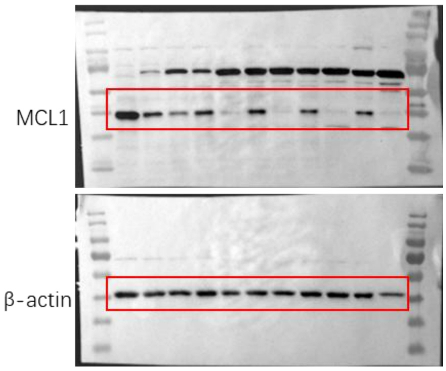

Fig 4F MOLM13

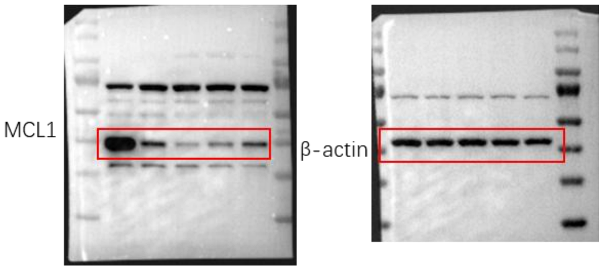

Fig 4G MOLM13

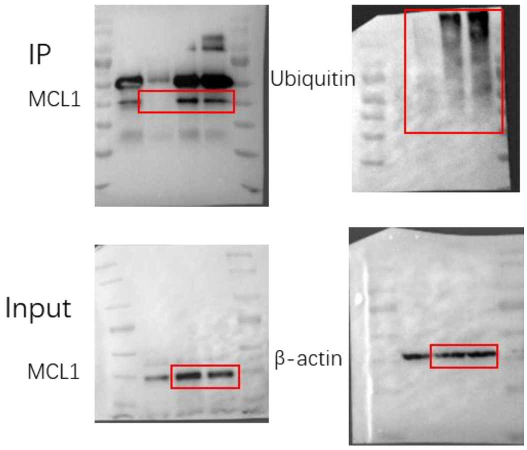

Fig 4H THP-1

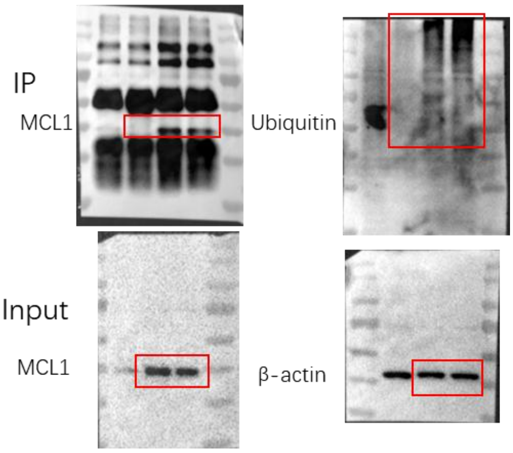

Fig 4J

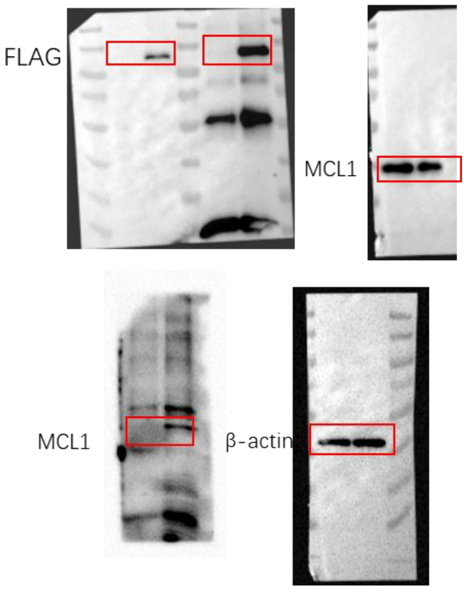

Fig 4K

MOLM13

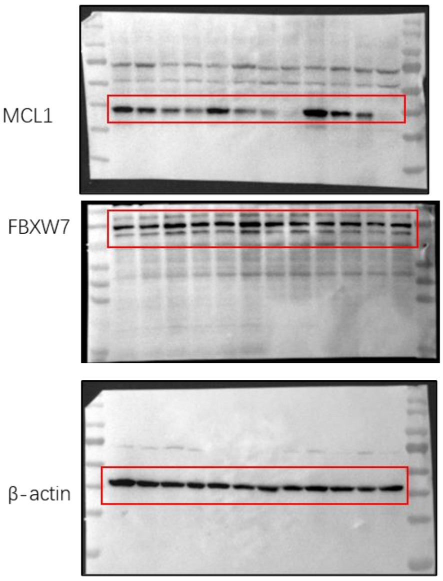

Fig 4L

THP-1

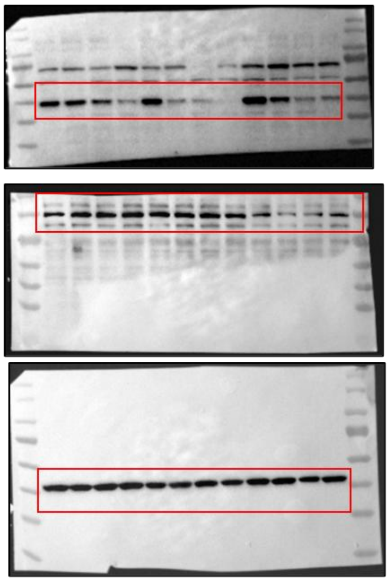

Fig 4O

MOLM13

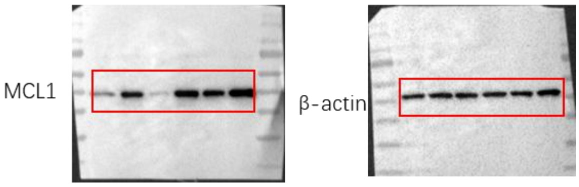

Fig 5A MOLM13

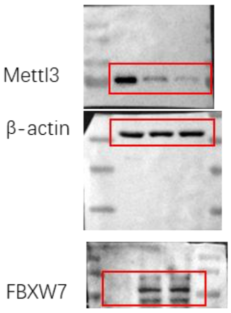

Fig 5B THP-1

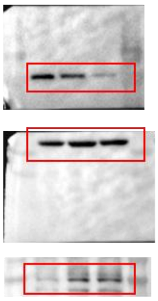

Fig 5C MOLM13

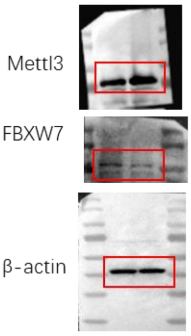

Fig 5D THP-1

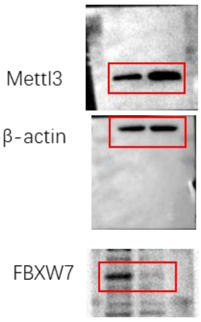

Fig 5H MOLM13

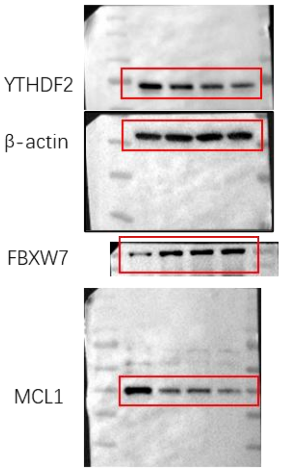

Fig 5I THP-1

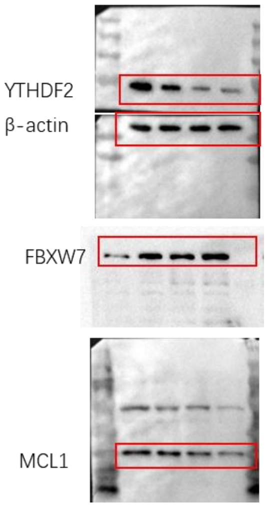

Fig 6C

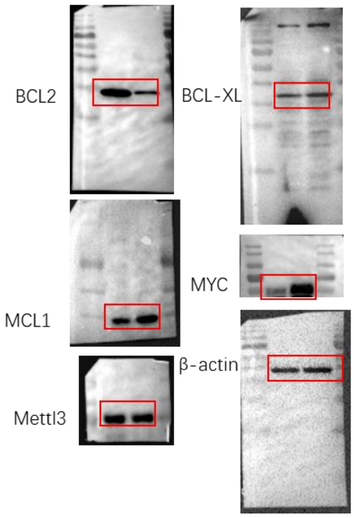

Fig 6D

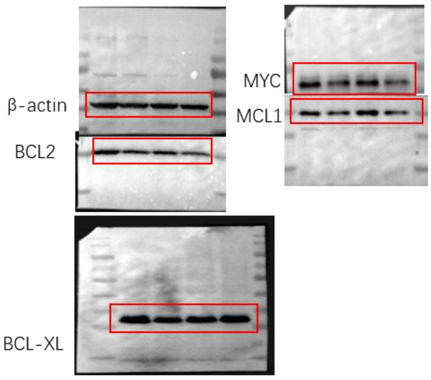

Fig 6E

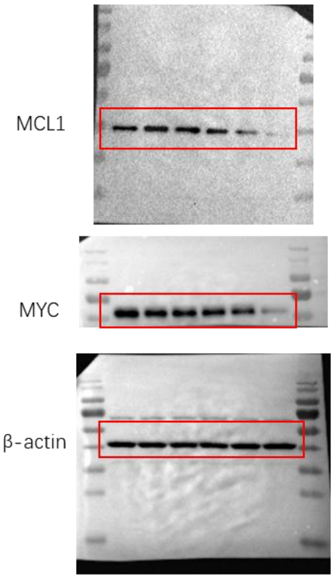

Fig 6I

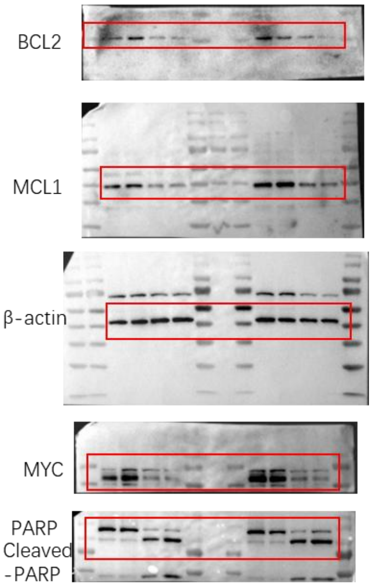

Fig 7B MOLM13

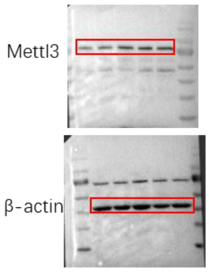

Fig 7C THP-1

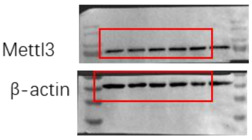

Fig 7D MOLM13 THP-1

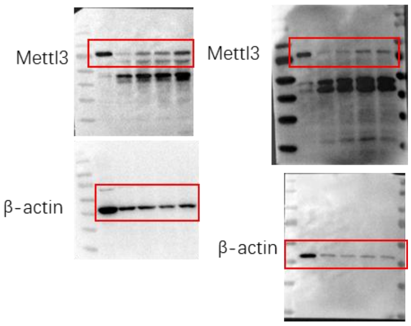

Fig 7F MOLM13

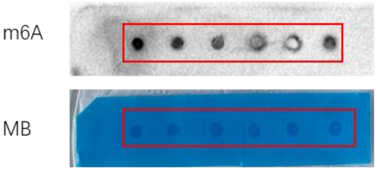

Fig 7G THP-1

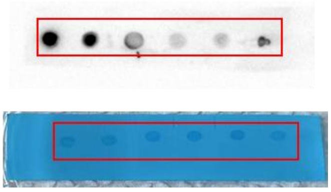

Fig 7I MOLM13

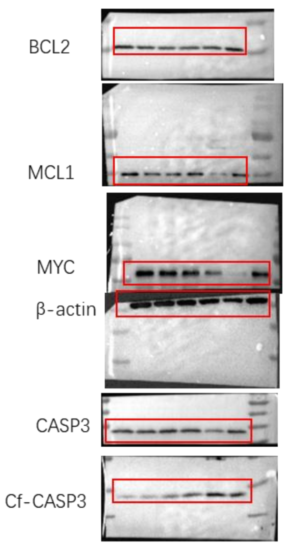

Fig 7J THP-1

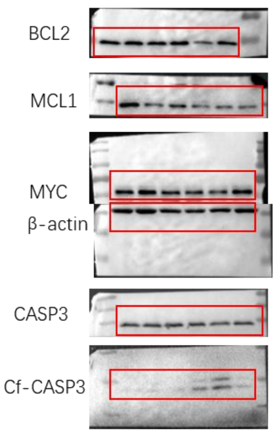

Fig 7M MOLM13

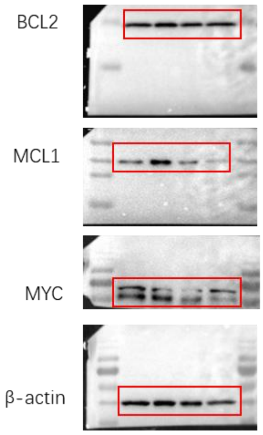

Fig 7N THP-1

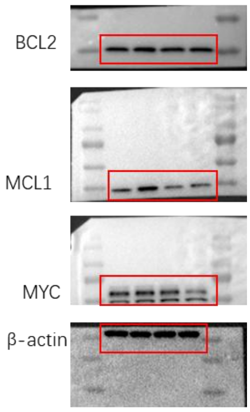

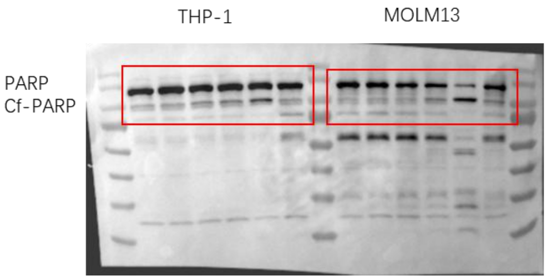

Fig S2A MOLM13 THP-1

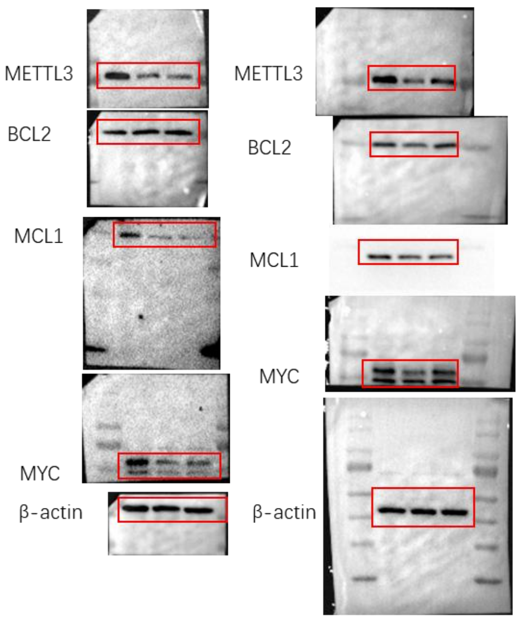

Fig S2B MOLM13 THP-1

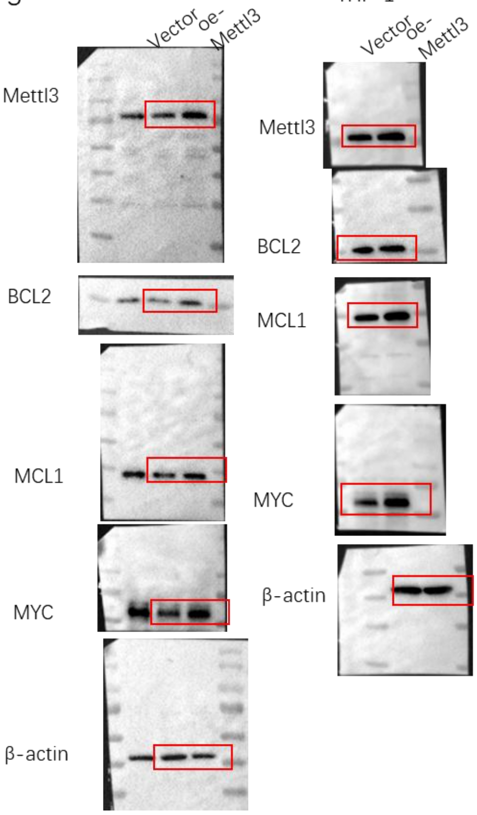

Fig S4B

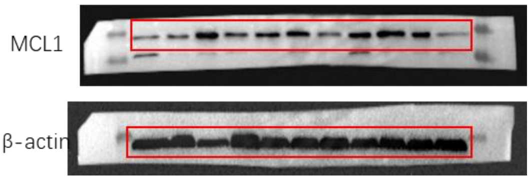

Fig S7A

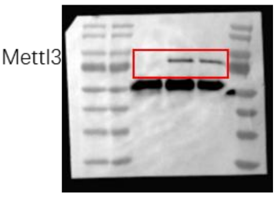

Fig S8A THP-1

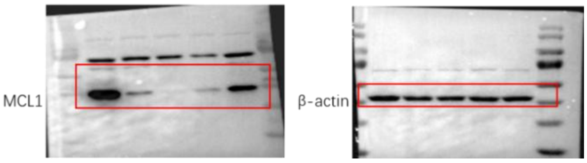

Fig S8E MOLM13

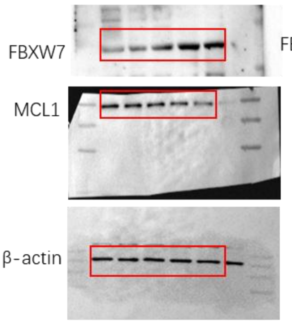

Fig S8F THP-1

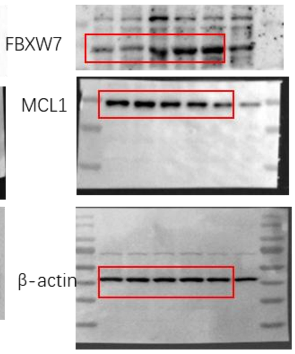

Fig S8G MOLM13

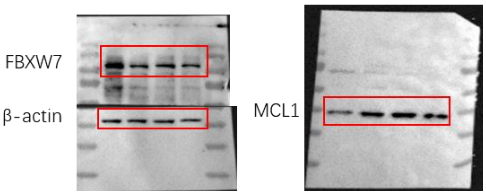

Fig S8H

THP-1

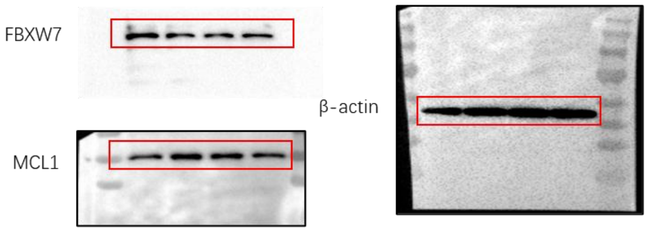

Fig S8I

THP-1

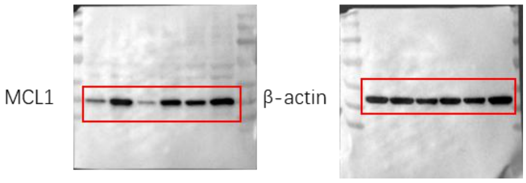

Fig S10B

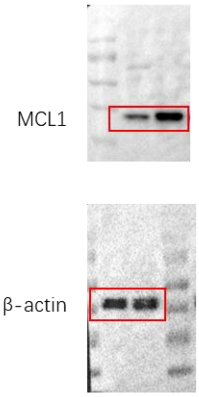

Fig S10F

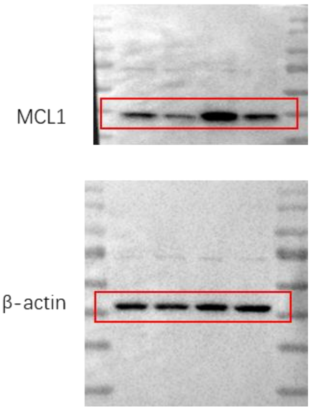

Supplement: Supplementary file 2 — Western blot original data [file 41419_2025_7560_MOESM2_ESM.pdf]
